# Proactive Maintenance of Pump Systems Operating in the Mining Industry—A Systematic Review

**DOI:** 10.3390/s25082365

**Published:** 2025-04-08

**Authors:** Sylwia Werbinska-Wojciechowska, Rafal Rogowski

**Affiliations:** Faculty of Mechanical Engineering, Wroclaw University of Science and Technology, Wyspianskiego 27, 50-370 Wroclaw, Poland; rafal.rogowski@pwr.edu.pl

**Keywords:** predictive maintenance, condition-based monitoring, pump diagnosis and prognosis, pump systems, the mining industry, systematic review

## Abstract

Recently, there has been a growing interest in issues related to mining equipment maintenance, with particular focus on pumping systems’ continuous operation. However, despite wide applications of pump system maintenance in a wide range of industries, such as water and wastewater, aviation, petrochemical, building (HVAC system), and nuclear power plant industries, the literature on maintenance of pump systems operating in the mining industry still needs development. This study aims to review the existing literature to present an up-to-date analysis of maintenance strategies for mining pumps, with a particular focus on proactive maintenance approaches. Key aspects considered include predictive diagnostics and prognosis, health status monitoring, maintenance management, and the integration of intelligent mining systems to enhance operational reliability and efficiency in harsh mining environments. The proposed methodology includes a systematic literature review with the use of the Primo multi-search tool, adhering to the Preferred Reporting Items for Systematic Reviews and Meta-Analyses (PRISMA) guidelines. The selection criteria focused on English studies published between 2005 and 2024, resulting in 88 highly relevant papers. These papers were categorized into six groups: (a) condition/health status monitoring, (b) dewatering system operation and maintenance, (c) health diagnosis and prognosis, (d) intelligent mining (modern technologies), (e) maintenance management, and (f) operational efficiency and reliability optimization. A notable strength of this study is its use of diverse scientific databases facilitated by the multi-search tool. Additionally, a bibliometric analysis was performed, showcasing the evolution of research on pump maintenance in the mining sector over the past decade and identifying key areas such as predictive diagnostics, dewatering system optimization, and intelligent maintenance management. This study highlights the varied levels of research and practical implementation across industries, emphasizing the mining sector’s unique challenges and opportunities. Significant research gaps were identified, including the need for tailored diagnostic tools, real-time monitoring systems, and cost-effective maintenance strategies specific to harsh mining environments. Future research directions are proposed, focusing on advancing predictive maintenance technologies, integrating intelligent systems, and enhancing operational efficiency and reliability. The study concludes with a detailed discussion of the findings and their implications, offering a roadmap for innovations in pump maintenance within the mining industry.

## 1. Introduction

The mining industry faces significant challenges driven by dynamic market demands, technological advancements, and evolving safety and environmental regulations. Effective machinery maintenance is a critical component in sustaining reliable and safe mining operations. Among the various systems used in mining, pumping stations play a pivotal role in water management and dewatering processes, particularly in underground mines where water intrusion poses substantial risks to operational safety and productivity. The ability to maintain the durability and reliability of these systems is vital for ensuring continuous mining operations and preventing costly downtime [1]. In addition, maintenance costs constitute a significant portion of total mining expenditures. According to industry reports, including data from the Empowering Pumps & Equipment portal [2], maintenance expenses can account for 35% to 50% of total mining project costs. Unplanned downtime of critical systems, such as pumps, can lead to severe financial consequences due to production losses and increased repair expenses. Therefore, adopting effective maintenance strategies, particularly transitioning from reactive to proactive maintenance, is essential for reducing operational risks and improving cost efficiency.

Mining operations must navigate a variety of factors that impact the performance of pumping systems. Harsh operating conditions, including abrasive slurries, corrosive substances, and fluctuating mechanical loads, accelerate wear and degradation of pumps and associated equipment. Maintenance strategies must, therefore, address these challenges through a combination of predictive and preventive practices. Predictive maintenance, leveraging technologies such as vibration analysis, thermography, and real-time data analytics, has become increasingly important in identifying potential failures before they result in breakdowns. This proactive approach minimizes unplanned downtime, reduces repair costs, and enhances overall system reliability [3].

Preventive maintenance remains a cornerstone of pumping system upkeep, focusing on routine inspections, scheduled servicing, and the timely replacement of components. For instance, tasks such as inspecting seals, lubricating bearings, and cleaning filtration systems help mitigate common wear-related issues. However, the complexity of maintaining large-scale and interconnected pumping systems in mining requires careful planning and effective resource management. Companies must develop comprehensive maintenance schedules tailored to the specific demands of their equipment and operating environment [4,5].

Design choices and material selection also influence the reliability of mining pumping systems. Pumps exposed to abrasive media benefit from wear-resistant materials, while corrosive environments necessitate corrosion-resistant coatings and components. Optimizing these design elements alongside robust maintenance practices improves the equipment’s resilience, ensuring better performance and extended service life [4]. Moreover, advancements in digital technologies, including the Internet of Things (IoT) and data-driven diagnostics, are transforming maintenance management by enabling remote monitoring and predictive analytics [6].

Another significant factor in maintaining mining systems is the competency and training of maintenance personnel. A skilled workforce equipped with the knowledge and tools to perform diagnostics, repairs, and proactive interventions is essential. Developing a strong maintenance culture, reinforced by regular training and adherence to safety protocols, enhances both the effectiveness and safety of maintenance activities. Organizations must prioritize continuous improvement to remain adaptive to new technologies and changing operational requirements [3,7].

Additionally, the necessity for a quick response in case of failures and unforeseen situations poses a significant challenge in mining operations. Pump failures and unplanned downtime can lead to severe operational disruptions, increased costs, and heightened safety risks. Therefore, mining companies must implement robust measures to minimize the likelihood of system breakdowns and ensure continuous operation. Employing modern maintenance technologies, such as predictive analytics, real-time condition monitoring, and automated diagnostics, significantly improves the responsiveness and efficiency of maintenance processes. These innovations enhance the reliability and availability of pumping systems while reducing maintenance costs and operational risks. Investments in advanced maintenance strategies and technologies strengthen the overall resilience of mining operations, ensuring sustainable performance, safety, and competitiveness in a demanding industry. In conclusion, addressing the challenges of mining system maintenance, particularly for pumping stations, requires a holistic approach that integrates predictive and preventive strategies, advanced technological solutions, and well-trained personnel. As mining operations evolve and the complexity of machinery increases, companies must invest in innovative maintenance practices to enhance reliability, reduce costs, and maintain a competitive edge in a demanding global market.

Recently, numerous studies and publications have emerged related to the mining sector, focusing on maintenance management and diagnostic strategies aimed at improving the reliability and operational efficiency of critical machinery (for a comprehensive review, see, for example, [8,9,10,11,12]).

Given the extensive range of maintenance strategies discussed in the literature—spanning from reactive maintenance to predictive maintenance—one of the key decision-making challenges is selecting the most appropriate strategy that aligns with mining companies’ specific needs and operational capabilities [13,14]. Several studies have addressed this issue, including analyses of preventive maintenance and inspection policies [15,16,17]. The feasibility of implementing condition-based maintenance in the mining sector is explored in works such as [18,19,20,21], while predictive maintenance strategies and their implementation are reviewed in [6,22,23]. Moreover, numerous studies focus on predictive maintenance applications and diagnostic or early warning methods, including fan failure prediction (e.g., [24]), gearboxes maintenance (e.g., [19]), bearings diagnostics (e.g., [25]), or mobile mining equipment maintenance (e.g., [26,27,28]). Additionally, research addressing the integration of Industry 4.0 technologies and artificial intelligence in mining equipment maintenance can be found in, e.g., [29,30,31].

When it comes to the problem of pump maintenance, the aspects of reliability and diagnostics are extensively explored, with examples provided in [32], where case studies on centrifugal and reciprocating pumps are presented. A review of predictive maintenance strategies for pump systems is available in works such as [33,34], although these studies primarily analyze heat pumps in combined heat and power plants. Additionally, the maintenance challenges of large-scale heat pumps are comprehensively summarized in [35].

Taking one step further, a search for English-language review publications in the Scopus database, using the keywords “*mining industry OR mine*” AND “*pump maintenance*” AND “*review* OR *state of the art* OR *current state*”, resulted in the identification of eight relevant records published between 2019 and 2023.

In work [10], the authors focus on the role of artificial intelligence (AI) and machine learning (ML) in improving predictive maintenance (PdM) strategies for machinery and equipment in the mining industry, which is crucial for continuous production but involves high costs and complexity. It provides a systematic review of current research, examining PdM methodologies, architectures, and models, as well as potential applications and challenges specific to the mining sector. The issues in the PdM area also examined in work [36]. The authors focus on the PdM in the context of Industry 4.0, highlighting its potential to reduce downtimes, lower costs, and enhance productivity through data-driven solutions in smart manufacturing. They provide a systematic review of PdM applications across various manufacturing sectors (including the mining sector), offering a comparative decision support map, insights into technology readiness, and a framework to guide the development of PdM strategies while addressing existing challenges. Another work, [37], reviews the current state of digital twin (DT) technology and its applications in the minerals industry, addressing challenges such as geological unpredictability, legacy system integration, and cybersecurity. It emphasizes the potential of combining immersive visualization with real-time spatial graphics to enhance the usability and acceptance of DTs, particularly in operational scenarios, and highlights the need for further research in this area. In addition, there are distinguished reviews that focus on the specific areas on maintenance (e.g., [38]) or type of objects—(e.g., [39,40]). The article [38] reviews the importance of condition-based monitoring for induction motors (IMs) to reduce operational and maintenance costs through early fault detection, minimizing downtime and unexpected failures. It presents an overview of IM faults, diagnostic schemes, and monitoring techniques, emphasizing the potential of non-invasive methods for automating maintenance scheduling and predicting failures in industrial applications. Article [40] focuses on wear issues in hydraulic components of hydrostatic transmission systems, exploring solutions like thermal coating and surface treatments, including laser beam and plasma coatings, to extend the lifespan of pumps, valves, and cylinders. Meanwhile, article [39] reviews the use of vibration signal analysis for monitoring rotating components, discussing advancements in signal processing, AI-based diagnostics, and prognostics and identifying the need for better interpretability, experimental validation, and data reproducibility to facilitate real-time industrial applications. Together, these reviews provide a comprehensive perspective on modern approaches to equipment maintenance and reliability in industrial environments. However, despite the increasing recognition of the role of pumps in ensuring continuous material flow and water management in mining operations, there is a notable gap in comprehensive review articles specifically addressing maintenance strategies, diagnostic technologies, and automation potential for mining pumps. Only a few of the identified studies touch upon general trends in pump maintenance without a detailed examination of the mining sector’s unique operational constraints and evolving technological landscape. Some examples of mine-specific dewatering solutions can be found in [41], where a comparative analysis of basic dewatering systems used in coal mines in India highlights efficiency-related issues in these systems. This lack of focus is particularly striking in the context of Maintenance 4.0, where advanced data-driven techniques, predictive analytics, and real-time monitoring are revolutionizing maintenance practices.

Consequently, a systematic review of existing approaches, technological advancements, and research gaps in mining pump maintenance is necessary to provide a holistic understanding of proactive maintenance strategies. This includes an assessment of predictive diagnostic methods, cost-effective maintenance planning, and the application of real-time monitoring technologies. The findings aim to guide both future research and industrial practices in enhancing the reliability and efficiency of pump systems in the mining sector.

Following the above considerations, this study provides a comprehensive overview of academic research on the issues of pump system maintenance, with particular emphasis on the mining industry application field. The primary goal is to identify key research trends in this field and suggest potential future research directions. Additionally, based on the literature review, a framework for proactive maintenance in pump systems from the mining industry is developed. This framework outlines a systematic approach for improving the reliability and efficiency of pumping systems by integrating predictive maintenance techniques, real-time monitoring, and the application of advanced technologies such as IoT and AI. Consequently, this paper contributes to the existing knowledge on pump systems maintenance in three ways: (1) identifying the major research trends related to mining industry applications in the maintenance of pump systems; (2) outlining future research directions for the study of pump maintenance in the mining industry sector; and (3) developing a framework for proactive maintenance of pump systems in the mining industry.

Based on these objectives, the research questions are as follows:

RQ1: What is the state of the literature on mining equipment maintenance, with a particular focus on pump systems, between 2005 and 2024?

RQ2: What are the main research and knowledge gaps in pump systems maintenance, especially in the mining industry sector?

RQ3: What trends can be identified in proactive maintenance approaches, and how have they evolved over recent years in the mining sector?

RQ4: What scope should the framework for proactive maintenance of pump systems in the mining industry have?

This paper addresses the research questions posed above by employing bibliometric performance analysis and systematic analysis using the PRISMA method (Preferred Reporting Items for Systematic Reviews and Meta-Analyses) [42]. This approach is designed to summarize and pinpoint the key research areas within the identified application fields.

In summary, the article is structured into seven sections. Following the Introduction (Section 1), the Theoretical Background (Section 2) outlines the issues of pump systems in the mining industry with a particular view on the maintenance of pump systems. The Review Methodology (Section 3) details the primary methods used for the review, including the strategy for the literature search and the criteria used to assess the relevance of the analyzed documents. Section 4 presents the main findings of the systematic literature review for the selected papers within the six identified application fields. Section 5 then discusses the results related to these application fields, identifying gaps in the existing literature and knowledge. Section 6 introduces a framework for proactive maintenance in pump systems from the mining industry. The final section, Conclusions (Section 7), provides a summary of contributions, outlines limitations, and offers recommendations for future research.

## 2. Theoretical Background

The analysis of the literature review would be incomplete without a presentation of the theoretical background related to the issue under study. Within the framework of their research work, the authors focus on pump systems operating in the mining industry. Therefore, in the next subsections, issues related to the specificity of such systems, focusing on mine dewatering systems in the investigated industry and currently used maintenance strategies for mining pumps, will be discussed.

### 2.1. Pump Systems in the Mining Industry

The mining industry involves discovering and extracting naturally occurring minerals from the earth [43]. Materials such as coal, metals, oil shale, rock salt, chalk, stone, and gravel are all extracted in mining. The recovery of these raw materials is based on the economic viability of investing in labor, mining tools, and energy to run mining operations, refining, and transport [44].

The exploitation of mineral deposits varies in terms of the way the work is carried out. Indeed, we may identify two main types of mining: surface mining and underground mining [45]:

- Surface mining activity means that all the work related to the extraction of raw materials takes place on the surface, and the entire process of extracting minerals is based on the discovery of successive layers of the deposit of raw material.
- Underground mining, also known as subsurface mining, consists of digging or blasting tunnels and shafts into the earth’s crust to reach buried orebodies. Ore deposits and tailings are brought to the surface to be refined by onsite processing plants.

**Figure 1:**
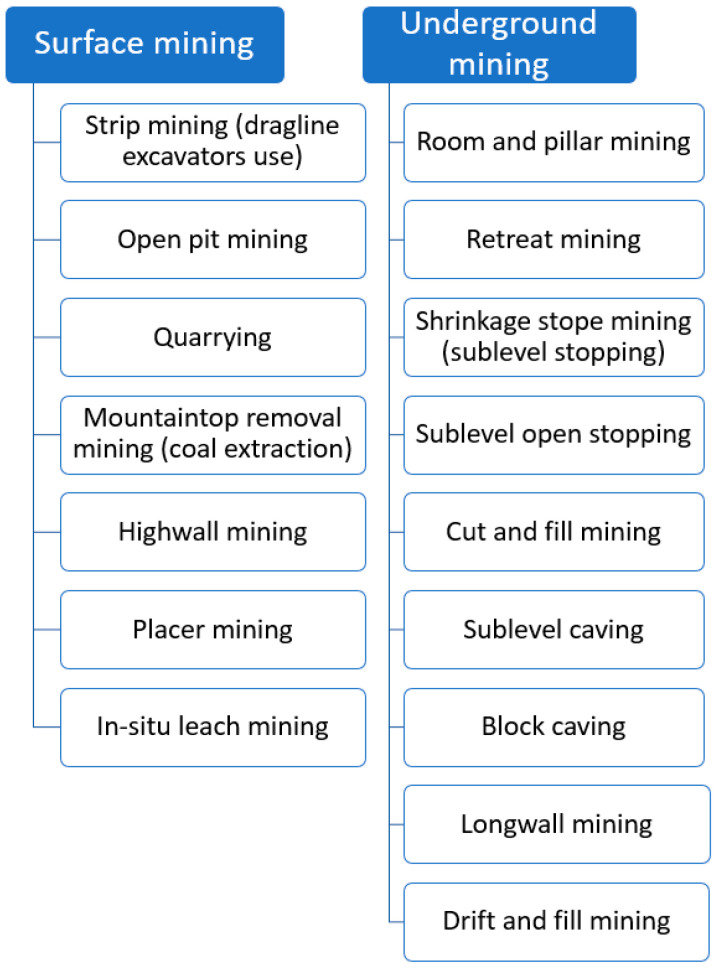
Surface mining and underground mining methods. Source: own contribution based on [46].

In addition, both types of mining activities use different methods for raw materials extraction (Figure 1). Their use results in various operational and maintenance problems that occur in mining companies.

Going one step further, it is evident that underground as well as surface mining of various mineral deposits involve the presence of water [41]:

- Water that is needed for technological processes performance;
- Water that remains after technological processes performance;
- Water as an inflow to mine working areas caused by ore exploitation.

Water can also enter the pits due to rainfall in the surface mines.

The demand for water needed for operations performance and the removal of surplus water is one of the main problems in the mining industry. This fact enforces the maintenance of dewatering systems at a high level to ensure the possibility of conducting mining operations. In addition, the problem becomes more complex due to the presence of different dewatering systems in the mine:

- Horizontal dewatering system;
- Main (vertical) dewatering system;
- Technological water system;
- Other water systems, such as a fire water system or an air-conditioning system.

The authors present an example of a mine shaft dewatering system’s operation and maintenance in [47], introducing the main challenges in this area.

**Figure 2:**
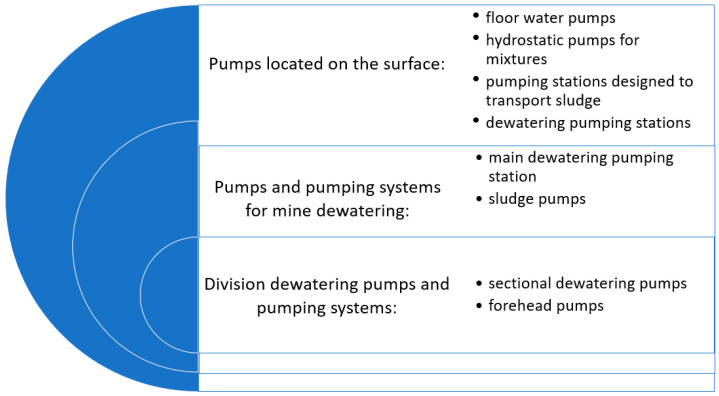
Pumps and pumping systems used in the mining industry. Source: own contribution based on [47,49,50].

The proper performance of water systems in the mine is based on using various pump systems. Pumps belong to a group of working machines called fluid conveyors. Their task is to pump (move) liquids (or mixtures of liquids with solids) through the system (overcoming local and linear flow resistance). In addition, the pump’s tasks are also connected with lifting the liquid from a lower level to a higher level and pumping the liquid from a vacuum tank with a lower pressure to a compression tank with a higher pressure to create a high working pressure of the liquid (e.g., hydraulic drives). In the pumping process, the pump takes mechanical energy from the motor and transfers it to the liquid flowing through it via the working unit (piston, rotor, impeller, etc.) [48]. Indeed, following this short introduction, the first classification of pump systems operating in mine dewatering systems is presented in Figure 2. The classification focuses on the application area in the mining industry—mine dewatering.

Water flowing out of the underground, along with technological water used, for example, in the process of drilling boreholes, is accumulated in the pits of forehead pumping stations. These are small pumping stations located in the mine workings, near the mining fields. Each mining field has at least one forehead pumping station. Water from the forehead pumping stations is transported to local pumping stations built at the main mine roadways. Local pumping stations have small settling tanks where water is collected and pre-cleaned of solids by sedimentation. Divisional pumping stations collect water from local pumping stations from a given mining district. They have settling tanks to ensure adequate water retention and sedimentation. The final stage of the dewatering process is the main pumping stations located at the shafts. Their task is to collect and drain water from the entire mine. Their tanks have retention, allowing them to collect water, purify it thoroughly, and pump out the daily inflow [51].

In addition, main dewatering systems for underground mine workings can be divided into the following categories [47]:

- Single stage;
- Multi-level;
- Indirect;
- Direct.

Moreover, according to [52,53,54,55,56], we may define three types of pumps used in the mining industry with their applicability:

- Slurry pumps, widely used in mining to transport abrasive mixtures of solids and liquids. They are prone to wear and tear due to the abrasive nature of the materials they handle.
- Submersible pumps, commonly used for mine dewatering. They are preferred for their ease of installation and maintenance.
- Centrifugal pumps, used for various applications, including dewatering and slurry transport. They require careful selection and maintenance to ensure longevity and efficiency.

**Figure 3:**
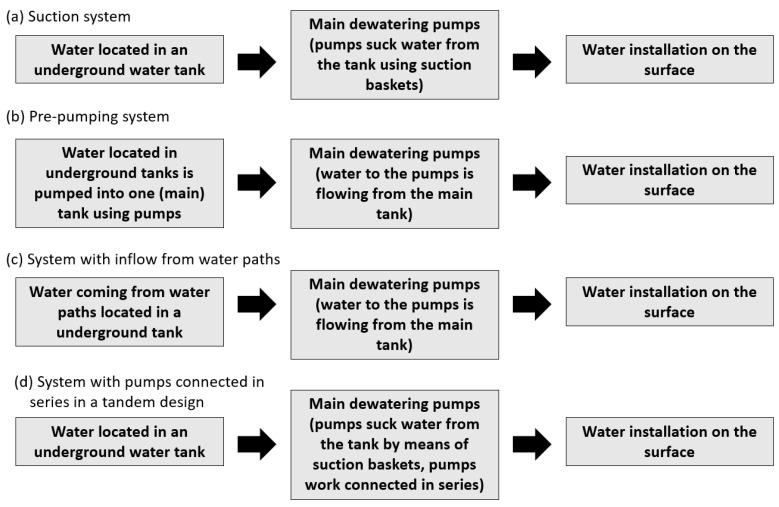
Single-stage dewatering systems. Source: own contribution based on [47].

Going step further, we can distinguish four single-stage dewatering systems (Figure 3). Systems (a) and (d) are characterized by the fact that the pumps are located above the liquid mirror. Pumps in such systems must be characterized by good cavitation properties. This condition forces the use of pumps with no more than 1500 rpm speeds. Pump systems of (b) and (c) are inflow systems that allow using pumps with lower cavitation properties. They may use pumps that reach 3000 rpm.

**Figure 4:**
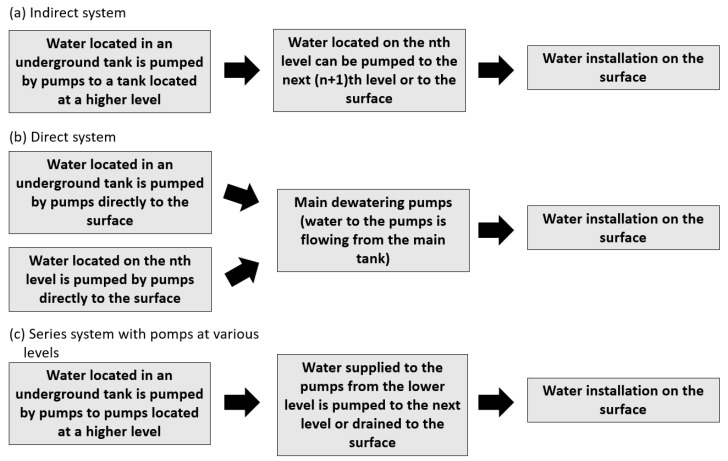
Multi-level dewatering systems. Source: own contribution based on [47].

Multi-level dewatering systems are characterized in Figure 4.

The selection of the right dewatering system in which the drainage pumps operate depends on a number of factors [57,58]:

- The number of levels on which mining is executed;
- The distance from the mining decks;
- Water inflow on each level;
- The irregularity of the inflows;
- The possibility of collecting water in the pits.

Going one step further, pumps can be classified into one of two main groups: dynamic (non-positive displacement) pumps and positive displacement pumps [59]. The first group of pumps uses the fluid velocity and the resulting momentum to generate the pumping power and move the fluid through the system. The second type of pump can operate by forcing a fixed volume of fluid from the inlet pressure section of the pump into the discharge zone of the pump [59,60]. In another work [61], the authors provide a more detailed classification of pumps based on three groups’ definitions: rotodynamic pump, positive displacement pump, and other pumps (impulse pump, gravity pump, steam pump, and valveless pump).

Pump systems are the second most important systems for the operation of a mine, following ventilation systems. They ensure the safety of workers and allow the mine to function. Therefore, these systems must be maintained at the highest possible efficiency to ensure operational continuity. Indeed, in the next subsection, pump maintenance issues are investigated.

### 2.2. Maintenance of Pump Systems

In mining, there are, among other things, mineral sludge, wastewater, kaolin slurries, and compacted sludge, which have to be dealt with by suitable pumping systems and equipment. Therefore, many types of pumps in the mining industry are designed for pumping clean and contaminated water and sludge with various muds. These pumps have to meet the requirements of each substance, and the safety of workers and the efficiency of their work depend on the quality of the pump used and the correctness of the maintenance processes carried out [62,63]. At the same time, machinery and equipment operating in the mining industry are also exposed to many factors related to the working environment, e.g., salts, dust, humidity, and high temperatures. In order to meet these requirements, these systems are designed in such a way that their sensitivity to these factors is as low as possible. Therefore, due to the difficult operating conditions, maintenance issues become particularly important in the case of such systems [41].

Pump systems, second only to ventilation systems in importance, are critical to mine operations. These systems ensure worker safety and enable continuous mine functionality. Consequently, maintaining pump systems at peak performance is essential for uninterrupted operations. Main dewatering pump stations must continuously remove water from mining excavations, regardless of failure conditions or production halts. Two independent energy sources must power such systems. Control systems for these pumps should allow both automatic and manual switching between units. Depending on system design and operational requirements, various parameters are monitored, including flow rate, water temperature, bearing temperature, supply voltage, motor current, lift height, and operating pressure. These measurements guide pump regulation and inform diagnostic actions [57].

#### 2.2.1. Pump Systems Reliability Issues

Enhancing the reliability of machinery operating in the mining industry requires a comprehensive understanding and identification of the root causes of failures, which form the basis for implementing predictive maintenance systems. Despite advancements in diagnostic methods, the current analysis and response to early failure symptoms often remain delayed, preventing the full realization of benefits offered by predictive maintenance strategies. Reactive maintenance, instead of proactive measures, contributes to increased operational costs and a lower utilization rate of machinery [63].

The reliability of pump systems largely depends on evaluating their electrical, mechanical, and electromechanical parameters. Condition assessment can be performed through two approaches: a limited-scope evaluation using diagnostic parameters from machine-installed sensors and a comprehensive, periodic global assessment. Local diagnostics involve monitoring technical conditions, operational parameters, and protective systems. Continuous online monitoring of parameters is essential to obtain a complete diagnostic picture. Data acquisition from operating equipment is facilitated by sensors and transducers selected based on the monitored circuit and parameters, such as pressure, temperature, and current draw. All collected data are archived in a central supervisory computer.

The mining industry employs a wide range of pump types, and accurately diagnosing failure sources requires a thorough analysis of the factors contributing to the malfunction. A critical aspect of pump diagnostics is determining the time of the first failure symptoms and tracking their progression over time. In facilities adhering to predictive maintenance principles, equipment condition forecasting is based on information obtained through diagnostic monitoring. An early step in this process involves defining threshold values that trigger preventive actions to avoid unplanned downtime [64].

Effective prevention and prediction of pump failures require a thorough understanding of both the pump’s internal construction and its operational environment. The primary components susceptible to failure include impellers, seals, bearings, shafts, and casings. Failures in these elements are often accompanied by identifiable signs of damage, such as the following [4,65,66]:

- Excessive temperature generated at seals;
- Rotor looseness;
- Corrosion of the casing or impeller due to turbulence or cavitation;
- Damage to rolling elements (bearings);
- Failures caused by improper use of the pump outside its design parameters.

Before the pump’s performance is visibly impaired due to component failure, early warning symptoms may appear within the system, signaling operational anomalies. Although these signals do not yet indicate a complete loss of functionality, their timely detection enables preventive maintenance actions. Diagnostic efforts should focus on observing physical phenomena produced by the system, emphasizing periodic or intermittent inspections to capture the moments when corrective or repair actions are necessary.

The key measurable parameters during pump operation include the following [47]:

- Temperature: excessive heating in areas experiencing high friction;
- Pressure: a drop in pump efficiency;
- Power consumption: increased power draw, indicating inefficiencies;
- Vibrations: an increase in overall vibration levels, with bearing failures often preceded by changes in vibration patterns. Excessive vibrations may result from pump misalignment or cavitation resonance between the pump, its base, or valves located in the suction or discharge lines;
- Leakage: monitoring seal integrity and bolted connections.

An increase in any of these parameters typically signals a deterioration in the pump’s technical condition.

Depending on the criticality and diagnostic capabilities of the system, condition monitoring can be performed manually by diagnostics specialists or through automated systems using information networks that collect data from the entire machinery fleet. All operational data are archived, and deviations from normal conditions are flagged to prompt maintenance interventions.

#### 2.2.2. Pump Systems Maintenance Issues

**Figure 5:**
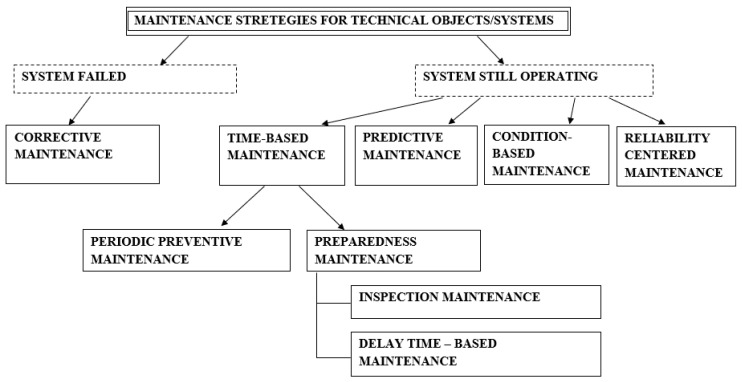
Technical system maintenance strategies. Source: own contribution based on [67].

The available literature offers numerous definitions of the term “maintenance” [67]. For the purposes of this research, the definition provided in the European standard PN-EN 13306:2010 [68] has been adopted. According to this standard, maintenance encompasses all technical, organizational, and managerial actions performed throughout the lifecycle of an asset aimed at retaining or restoring it to a condition in which it can fulfill its intended function. A comprehensive literature review on the maintenance of systems and equipment can be found in works such as [69,70,71]. A general classification of fundamental maintenance strategies is presented in Figure 5. A comprehensive review of the topic of maintenance modeling is provided in [67].

Effective pump maintenance involves a combination of traditional scheduled maintenance, condition monitoring, and predictive maintenance strategies. Leveraging technological advancements and best practices can significantly enhance pump reliability and operational efficiency, ultimately reducing costs and improving productivity.

Pump maintenance can be broadly categorized into preventive, corrective, and predictive maintenance strategies. Preventive maintenance refers to scheduled tasks performed to prevent potential issues, such as checking for leaks, cleaning the pump components, and replacing seals and bearings before they fail. On the other hand, corrective maintenance is carried out after a malfunction or failure occurs, addressing the issue at hand to restore the pump’s functionality. Predictive maintenance, the most advanced of the three, relies on monitoring systems and sensors to predict potential failures based on real-time data, such as vibration, temperature, and pressure readings. This type of maintenance is designed to minimize unplanned downtime by identifying issues before they lead to significant failures.

**Figure 6:**
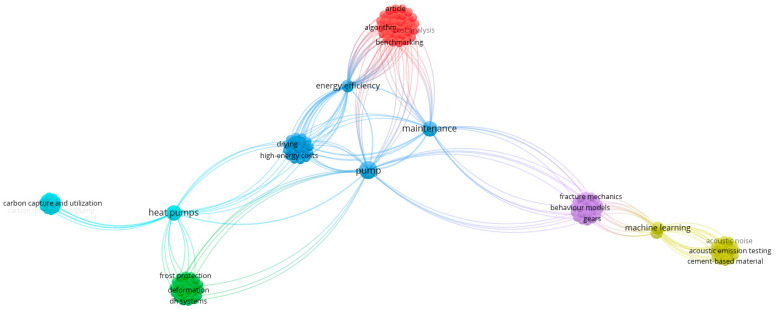
Mapping of the keywords that have occurred in the selected publications at least once—the Scopus database. Source: own development using VOSviewer software [72].

A significant body of literature is dedicated to pump maintenance, with numerous review papers addressing various aspects of the field. A preliminary literature analysis on pump maintenance was conducted using the Scopus database. A search for the term “pump maintenance” in article titles, abstracts, and keywords yielded 7252 results. To ensure relevance, the search was limited to subject areas such as engineering, energy, environmental science, computer science, mathematics, and business management and accounting. A keyword co-occurrence analysis based on using VOSviewer software ver. 1.6.18 of the obtained results allowed defining six main thematic clusters (Figure 6).

The first cluster—the largest cluster (red one, 31 keywords)—mostly focuses on the aspects of pumping scheduling and operational optimization. The second cluster (green one, 24 keywords) strictly investigates interactions between infrastructure, particularly railways, and thermal conditions. The third cluster (blue one, 23 keywords) focuses on improving the energy performance of pumps. The yellow cluster (22 keywords) develops the possible usability of AI-based methods in health monitoring. The next cluster (violet one, 22 keywords) is connected with the aspects of assessing and mitigating risks associated with failures of different types of pumps, and the last cluster (light blue, 15 keywords) is mostly focused on performance analysis in relation to typical faults of heat pumps.

The investigation of the reviewing literature in this area may extend this initial analysis. These works often focus on methods for improving maintenance strategies, such as the development of more accurate diagnostic tools, the integration of predictive maintenance technologies, and the analysis of failure modes and their causes. Many reviews also examine case studies where pump maintenance practices have been optimized, offering valuable insights into real-world applications and best practices. The growing interest in sustainability and the need for energy-efficient solutions has further fueled research in this area, as maintaining pumps in an optimal state can directly impact an organization’s environmental footprint.

The literature also highlights the importance of condition-monitoring techniques and the role of digitalization in modern pump maintenance. With advancements in sensor technologies and data analytics, pumps can now be monitored in real time, allowing for immediate corrective action when necessary. These innovations have led to intelligent pump systems that can self-diagnose and predict future maintenance needs, significantly reducing the likelihood of unexpected failures. Such systems are increasingly used in industries that rely on high uptime, such as power generation, water treatment, and mining. A summary of recent reviewing articles that focus on pump maintenance issues is presented in Table 1.

The articles presented in the table, focusing on reviewing pump maintenance issues, demonstrate that the topic has been extensively analyzed from various perspectives, including preventive strategies, predictive techniques, and technological advancements. These reviews highlight the importance of pump maintenance in ensuring operational efficiency and reducing downtime across multiple industries. However, there is a notable gap in the literature regarding review articles specifically focused on the mining industry sector. Given the critical role of pumps in mining operations, a comprehensive review tailored to this sector could provide valuable insights into industry-specific challenges, maintenance practices, and opportunities for improvement in this demanding environment.

Despite the lack of review articles, numerous studies are dedicated to pump maintenance in the mining sector, addressing key challenges such as abrasive wear, high-pressure demands, and energy efficiency. These works provide valuable insights into maintenance practices and solutions tailored to the unique conditions of mining operations. In the maintenance of the pump systems within the mining industry, two primary approaches are currently employed: the “run-to-failure” or corrective maintenance (CM) strategy and the preventive maintenance (PM) strategy [74]. The CM approach involves repairing or replacing components only after a failure occurs, leading to significant financial losses, downtime, and potential safety risks. In contrast, the PM strategy, particularly time-based preventive maintenance, may result in the premature replacement of components simply because their scheduled service life has ended, even if they are still functional. This method can be inefficient due to parts, labor, and service time costs. Additionally, unexpected failures can still occur between scheduled maintenance intervals, potentially leading to severe consequences [74].

An alternative approach gaining popularity in pump maintenance is condition-based maintenance (CBM). This strategy involves continuously monitoring key parameters that reflect the technical condition of a pump and its components using diagnostic tools [84,85]. CBM enables maintenance to be performed just before a failure occurs, improving efficiency by focusing on fault detection, degradation tracking, and failure prediction. Unlike CM or traditional PM, CBM emphasizes proactive monitoring of wear and tear to optimize maintenance activities. Studies such as [86,87,88] highlight its effectiveness in enhancing reliability.

Another increasingly applied strategy is predictive maintenance [89,90], which uses advanced diagnostic technologies to analyze the operating conditions of pumps and predict future failures. Continuous condition monitoring detects early signs of wear, allowing for timely interventions such as adjusting operating settings or improving lubrication [32]. PdM techniques commonly include thermal imaging, vibration analysis, and acoustic measurements [91]. Research on predictive maintenance of pumps, including fault detection and diagnosis, is presented in works like [22,92], while comprehensive reviews on fault diagnosis methods for pumping systems are discussed in [61,79]. Despite this, limited research has been conducted specifically to address maintenance challenges for mining pumps [65].

In summary, interest in pump maintenance in the mining industry is steadily growing, as confirmed by the increasing number of studies addressing the unique challenges of this sector. While traditional approaches like corrective maintenance (CM) and preventive maintenance (PM) remain widely used, their limitations in terms of efficiency, cost, and reliability underscore the need for alternative strategies. Condition-based maintenance (CBM) and predictive maintenance are emerging as promising solutions, leveraging advanced diagnostic tools and continuous monitoring to enhance operational efficiency, minimize downtime, and predict failures. Despite the growing body of research on pump maintenance across various industries, there is a notable lack of comprehensive reviews specifically focused on the mining sector.

This article aims to fill this gap by offering a detailed synthesis of current knowledge on maintenance practices for pumps in mining operations. It addresses two critical gaps in the literature:

- Lack of comprehensive reviews on mining-specific pump maintenance: While numerous studies broadly explore pump maintenance, few focus on the unique environmental and operational challenges the mining industry faces. This review provides a structured and in-depth analysis tailored to these conditions.
- Limited exploration of advanced maintenance strategies in mining: The application of CBM and PdM in mining pump systems remains underexplored. This article highlights the potential of these approaches to improve reliability and efficiency in harsh mining environments and proposes future research directions to optimize their implementation.

By addressing these gaps, this review aims to advance the understanding of pump maintenance in the mining industry and provide a foundation for developing more effective and sustainable maintenance practices. Additionally, this article will propose a framework for proactive maintenance in pump systems tailored to the unique conditions of mining operations, offering practical guidelines for improving maintenance strategies and minimizing downtime. This framework will be valuable for enhancing reliability, efficiency, and cost-effectiveness in mining pump systems.

## 3. Review Methodology

This section presents the key assumptions and steps undertaken in the systematic literature review (SLR) conducted for this study. An SLR aims to systematically identify, evaluate, and synthesize all relevant research pertaining to a specific research question, topic, or phenomenon of interest [93]. It is widely recognized as a rigorous scientific methodology that adheres to standardized procedures for designing, conducting, and reporting research processes [94,95].

**Figure 7:**
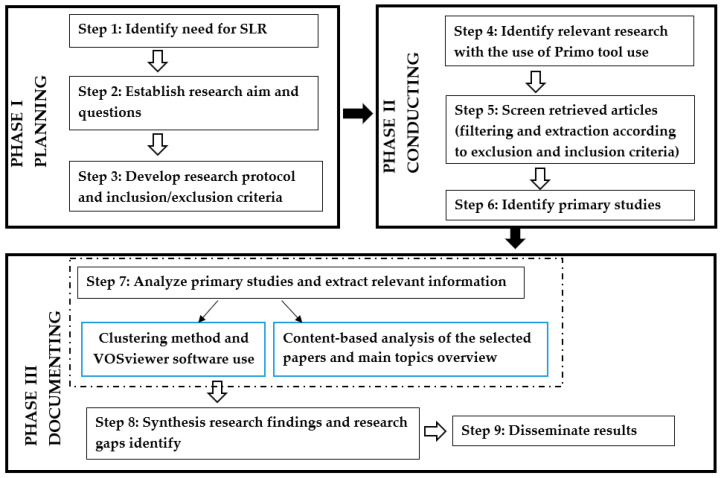
Research framework and methods/tools used for systematic literature review. Source: own contribution.

The primary objective of the review was to explore the main research trends and identify gaps in pump maintenance in the mining sector. The review process followed established guidelines outlined in [94,96,97] and was conducted in alignment with the PRISMA framework [98,99]. The SLR methodology employed by the research team consisted of nine steps, grouped into three phases: planning (three steps), conducting (three steps), and documenting (three steps), as shown in Figure 7.

The following subsections provide a detailed discussion of the research activities carried out during each of these three phases.

### 3.1. Planning of the SLR Study

This section outlines the key steps in this SLR study’s planning phase. The primary aim of the research is to review, classify, and synthesize the existing literature on pump maintenance in the mining industry based on a thematic analysis of relevant studies. The planning process’s first step involved defining the review’s main objectives. These objectives include (a) establishing a comprehensive understanding of maintenance strategies for pumps used in mining operations, (b) identifying key knowledge gaps and research opportunities in this field, and (c) highlighting trends and challenges to guide future research efforts. To achieve these objectives, a set of research questions was formulated to structure the review (step 2). These questions, presented in the Introduction section, were designed based on an in-depth analysis of existing studies on pump maintenance strategies, diagnostic and prognostic approaches, and the unique challenges associated with mining operations presented in Section 2. The preliminary review also emphasized the need to address the lack of a comprehensive framework tailored to the proactive maintenance of pumps in the mining sector.

Additionally, the research questions and the initial literature analysis informed the development of the study’s research framework, the selection of appropriate tools and methods, and the establishment of inclusion and exclusion criteria for the review process. This systematic approach ensures a focused and rigorous analysis of the relevant literature while addressing the identified research gaps (step 3).

### 3.2. Conducting the SLR Study

The second phase of the performed methodology includes steps 4, 5, and 6, which are discussed in detail below.

#### 3.2.1. Collection of Publications for Review

The first step involved conducting the literature search process, utilizing the Primo multi-search tool [100]. Primo allows users to explore a library’s collection of resources through keyword searches, with various filters available to refine the results. This approach enables the development of search strategies that incorporate resources from multiple scientific databases, including Scopus, Web of Science, and ScienceDirect, as well as databases from publishers such as Elsevier, Wiley, and Springer. The literature search was carried out over a one-month period, specifically from 20 December to 30 December 2024.

The search strategy utilized English terms combined with the Boolean operators AND and OR to form a comprehensive search query. Keywords related to digital twins, maintenance, and transportation aspects were selected for this purpose. Maintenance-related terms were identified based on existing review articles [10,34,79,101]. To ensure thoroughness, the keyword selection process incorporated both contemporary and historical terminology pertinent to pump maintenance in relation to the mining sector. This approach ensured relevance to both the research scope and historical developments in the field.

An initial broad search strategy was implemented, which included examining related terms (e.g., “dewatering system”, “maintenance AND mining industry”, “proactive maintenance AND pump systems”) before finalizing the keyword list. Ultimately, the selected keywords were chosen to achieve the broadest possible coverage of pump maintenance research within the selected industry sector context. The final search engine configuration includes the following keywords:
(ALL = (pump maintenance)) AND (ALL = (pump condition OR pump monitoring OR pump prognosis OR pump diagnosis OR pump fault)) AND (ALL = (mining industry))

Based on the presented criteria, the initial search allowed for the identification of 1142 scientific papers, which were later analyzed in the screening process (Section 3.2.2).

#### 3.2.2. Screening of Collected Publications

The screening process was crucial for identifying studies relevant to full-text analysis. Initially, the search was narrowed to include papers published between 2005 and 2024, ensuring a focus on recent advancements in the field. To ensure the quality of the selected papers, additional evaluation criteria were applied, including an analysis of the research methodology, the diagnostic tool quality, and the consistency of results with industrial requirements. The quality assessment was conducted based on the guidelines of the systematic review protocol (PRISMA), which also involved evaluating the reliability of sources and the transparency of results. Papers that did not meet these criteria were excluded from further analysis. Specifically, studies that lacked clear results or employed methodologies unsuitable for pump diagnostics in mining were excluded.

To thoroughly evaluate each study’s relevance to the topic of pump maintenance in relation to the mining industry, two primary inclusion criteria were applied: full-text availability and publication in English. Full-text access was essential for a detailed review of each study’s methodologies, findings, and alignment with the research focus. Limiting the selection to English-language publications ensured consistent interpretation and a comprehensive understanding of the studies. Based on these criteria, 375 papers were excluded from further consideration.

The screening process aimed to filter out studies unrelated to the research topic and was carried out in two steps. In the first step, all authors reviewed the titles and abstracts of the identified records. Papers were included if their abstracts focused on DT applications in transportation systems, operations, and maintenance. The team then discussed their assessments in a research meeting, and in cases of disagreement, articles were retained for full-text analysis. Following this step, 390 papers remained for further evaluation, with duplicates and review articles subsequently removed.

In the second step, a full-text review was conducted to determine the paper’s relevance to the thematic scope. Studies were evaluated based on their contributions to understanding pump maintenance applications in mine dewatering systems, the maintenance methods and models applied, and condition monitoring, diagnosis, and prognosis. As in the first step, team members performed evaluations individually, followed by group discussions during research meetings. When disagreements arose, the team conducted a more detailed document analysis. This step led to the exclusion of 730 papers for various reasons, such as studies focused on maintenance issues in unrelated fields like medical applications (pump fusions).

In the second step of the screening process, the authors examined the papers in the full-text research. The main aim was to assess their relevance to the investigated thematic area. Papers were evaluated based on their contributions to understanding DT applications, methodologies used, and their implications for maintenance and operational practices in transportation systems. As in the first step, the research teams made the evaluation individually. Later, at research team meetings, we compared team members’ opinions. In case of discrepancies in assessing the paper’s suitability, the team members focused on a more detailed analysis of the full document. As a result, 302 papers were excluded for specific reasons. For example, the studies that describe maintenance issues, e.g., medicine applications, were excluded.

#### 3.2.3. Primary Studies Identification

Following the screening process, 88 publications were chosen for subsequent qualitative and quantitative analysis. A cross-sectional review of the selected papers was also performed to ensure a comprehensive and representative collection of the relevant literature [102]. Papers were excluded if they did not meet the inclusion criteria or did not contribute adequately to the topic of pump maintenance in mining operations. Additionally, studies lacking methodological rigor or applied diagnostic tools irrelevant to mining pump systems were excluded. These exclusions were based on an assessment of the study’s research design, the proposed diagnostic methods’ applicability, and the findings’ relevance to the mining industry. This approach confirmed that the identified studies collectively offer a thorough overview of the current state of research in the analyzed area.

**Figure 8:**
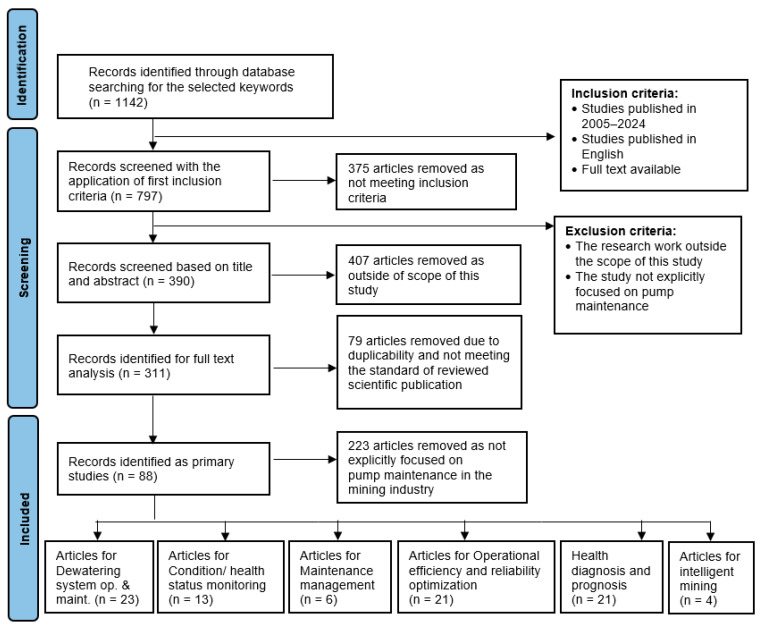
PRISMA-based flowchart of systematically selecting relevant studies in the analyzed research area. Source: own contribution based on [42].

The flow diagram illustrating the study selection process, aligned with PRISMA guidelines, is shown in Figure 8. Additionally, the PRISMA checklist detailing the methodology is provided in the Supplementary Materials (Table S1).

### 3.3. Documenting of the SLR Study

This phase corresponds to steps 7, 8, and 9 of the SLR process. As part of step 7, a bibliometric analysis was conducted.

Bibliometrics, a subfield of scientometrics, applies mathematical and statistical techniques to evaluate scientific activities. This analysis facilitates the exploration of networks formed around key representative keywords and highlights how citations, authors, affiliations, countries, and publications reflect the significance of specific topics in the research field. Notably, there has been a growing interest in bibliometric studies within the scientific community (see, e.g., [103,104,105,106,107]).

Following the analysis, the selected articles were organized, documented, and categorized using the Mendeley reference manager [108,109]. For primary content-based analysis, MS Excel Professional Plus 2019 and VOSviewer ver. 1.6.18 software were utilized [72]. The main findings were summarized using variables such as authors’ geographic locations or publication years.

VOSviewer, as described in [110], is a specialized tool for creating and analyzing bibliometric maps, allowing for a detailed examination of the results. The quantitative summary includes trend analyses and distributions by publication year and source. Using the functionalities of VOSviewer, bibliometric maps were created, focusing on keyword co-occurrence and relationships between leading authors. The findings are detailed in Section 4.

Step 8 involved synthesizing the research findings and aligning the results with the four defined research questions.

Finally, step 9 outlined the study’s limitations and suggested directions for future research. The outcomes of steps 8 and 9 are presented in Section 5 and form the foundation for developing the framework for proactive maintenance in pump systems from the mining industry (Section 6).

## 4. Results

This section includes the results of the conducted systematic review according to the defined research methodology (Figure 8). As a result, in the next subsections, bibliometric analysis and content-based analysis are presented.

### 4.1. Bibliometric Analysis

In the first step, a bibliometric analysis of already-selected publications for further research on the topic of pump maintenance in the mining industry was carried out. A total of 88 publications from the six subject areas analyzed were accepted for detailed analysis. The largest number of articles (23 papers) was in the area of dewatering system operation and maintenance (O&M). The number of analyzed publications in the other areas is as follows: health diagnosis and prognosis—21 papers; operational efficiency and reliability optimization—21 papers; condition/health status monitoring—13 publications; maintenance management—6 publications; and intelligent mining—4 publications.

The geographic distribution of authors and scientific institutions was also examined as part of the bibliometric analysis. The results reveal significant disparities in research activity worldwide. The United States and China lead in the number of publications, contributing approximately 23% and 21% of the analyzed papers, respectively. Poland and India also demonstrate strong research engagement, with 10 and 6 publications, respectively. Other countries show varying levels of contribution, including Africa and Hong Kong (5 papers each), as well as Germany, South Korea, and Australia (3 papers each).

**Figure 9:**
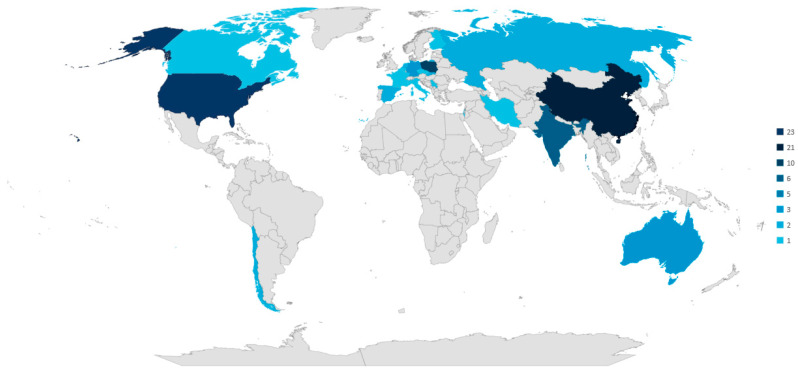
The number of papers by the location where the investigated study took place.

When viewed from a continental perspective, Asia emerges as the most active region, accounting for nearly 40% of all analyzed publications. Europe and North America also exhibit substantial research output in this domain, representing 26% and 24% of the total publications, respectively. In contrast, Africa contributes 5%, Australia 3%, and South America 2% of the total analyzed works. The regional distribution of authorship is illustrated in Figure 9.

The distribution of publications on pump maintenance across different countries and continents can be attributed to several key factors, including industrial activity, research funding, and technological development.

Countries like the United States and China lead in the number of publications due to their strong industrial bases, significant research and development investments, and major academic institutions collaborating with the industry. Both nations have extensive mining, energy, and manufacturing sectors, where pump maintenance is critical for operational efficiency and cost reduction. Additionally, government-backed initiatives and corporate research efforts drive innovation in predictive and proactive maintenance strategies.

Poland and India also show substantial contributions, which can be linked to their strong engineering and technical research traditions and the importance of heavy industry and energy production in their economies. Poland’s engagement in mining and manufacturing, along with its growing interest in Industry 4.0 technologies, supports active research in maintenance strategies. Conversely, India has a rapidly expanding industrial sector and is increasing investment in smart maintenance solutions, driving academic interest in the field.

**Figure 10:**
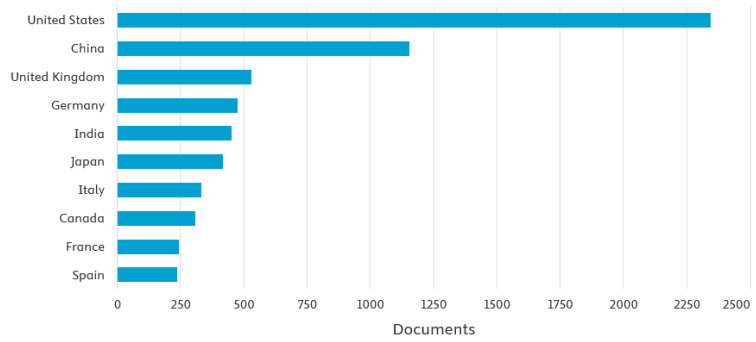
According to the Scopus database search via the keyword “pump maintenance” for the period 2000–2024, the number of documents by the location where the investigated study took place.

These trends are further confirmed by an analysis of publications in the Scopus database, where the keyword “*pump maintenance*” via a title/abstracts/keywords search yields 9439 publications for the period 2000–2024, showing a clear dominance of the United States and China in this research area (Figure 10).

The dominance of Asia as a region, with nearly 40% of all analyzed publications, aligns with its rapid industrialization, large-scale infrastructure projects, and focus on digital transformation in maintenance practices. Europe and North America also have significant research activity due to their well-established engineering sectors and advanced technological solutions in industrial maintenance.

The lower publication activity in regions such as Africa, South America, and Australia may be due to fewer research institutions focusing on pump maintenance, limited funding for maintenance-specific research, or a greater reliance on industry-led rather than academic-led innovation. However, given the importance of mining and energy industries in these regions, the potential for increased research in pump maintenance remains significant.

**Figure 11:**
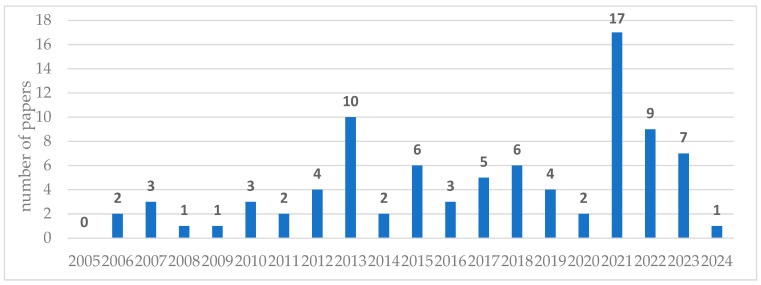
Distribution of publications by year.

In addition, this review brings together 88 publications that were published between 2005 and 2024. Figure 11 illustrates the distribution of the publications according to their publication year. As we can see, the distribution of publications by year is highly diverse. The highest number of publications falls within the period 2015–2023, with 59 publications accounting for 67% of all publications selected for analysis. Notably, the peak years were 2010 and 2021, with the most publications related to the studied area.

**Figure 12:**
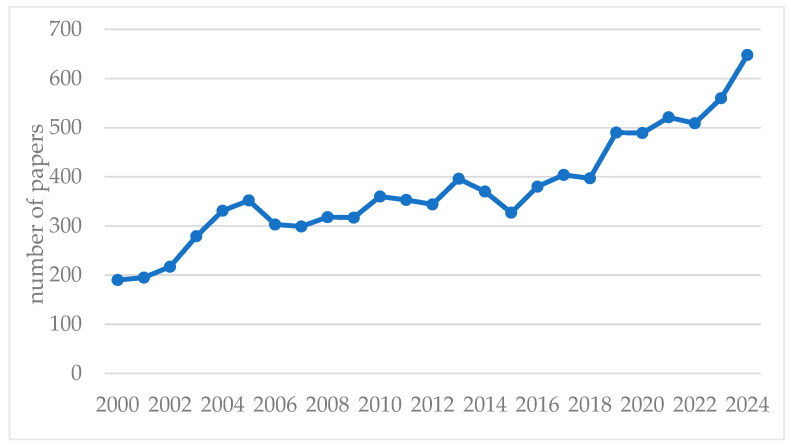
Distribution of publications by year, according to the Scopus database search via the keyword “pump maintenance” for the period 2000–2024.

Interestingly, since 2021, the number of these publications has been declining, reaching just a single entry in 2024. This downward trend may be attributed to a shift in research focus, where topics related to the predictive maintenance of pumps are being explored in a broader context without specifically emphasizing solutions dedicated to pumps within the mining industry. This conclusion is supported by the previously presented analysis of publications in the Scopus database, where the keyword “*pump maintenance*” via a title/abstract/keywords search for the period 2000–2024 was mentioned. According to the obtained results from the Scopus database, we can see a clear year-over-year upward trend (Figure 12).

**Figure 13:**
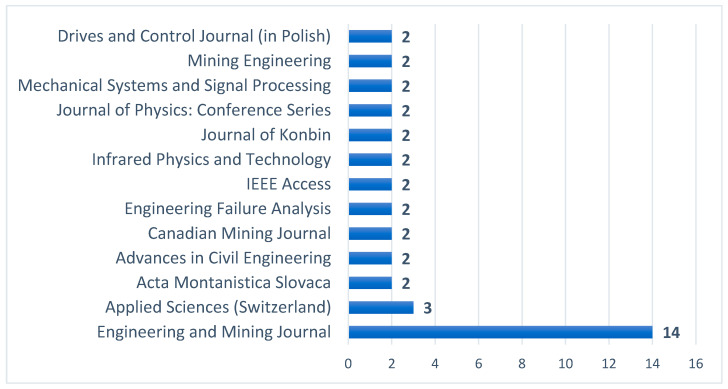
Number of publications with journal sources (for journals with at least two published articles out of the 88 articles analyzed).

Moreover, the analyzed articles were published across 44 different journals, reflecting the broad interest in the subject within various research communities. Figure 13 presents a graphical representation of journals that featured at least two publications related to the studied area. In total, 38 articles from 13 journals were considered in the analysis.

The *Engineering and Mining Journal* emerged as the most prominent publishing outlet, with 14 articles dedicated to the topic. *Applied Sciences* ranked second, featuring three relevant publications. The remaining journals each contributed two articles, indicating a more distributed but still notable engagement with the subject.

This distribution of publications highlights the interdisciplinary nature of research on pump maintenance and predictive strategies, spanning both industry-focused and broader scientific journals. The dominance of the *Engineering and Mining Journal* suggests a strong connection between pump maintenance research and the mining sector, where reliable equipment operation is crucial. Meanwhile, publications in *Applied Sciences* and other journals reflect the increasing academic interest in integrating new technologies, such as artificial intelligence and IoT, into predictive maintenance frameworks.

The many publications in these journals may also indicate their prestige and broad visibility within the scientific community in the analyzed research field. Authors may prefer to publish their work in widely read and respected journals, which enhances the impact of their research on the development of mining technologies.

**Figure 14:**
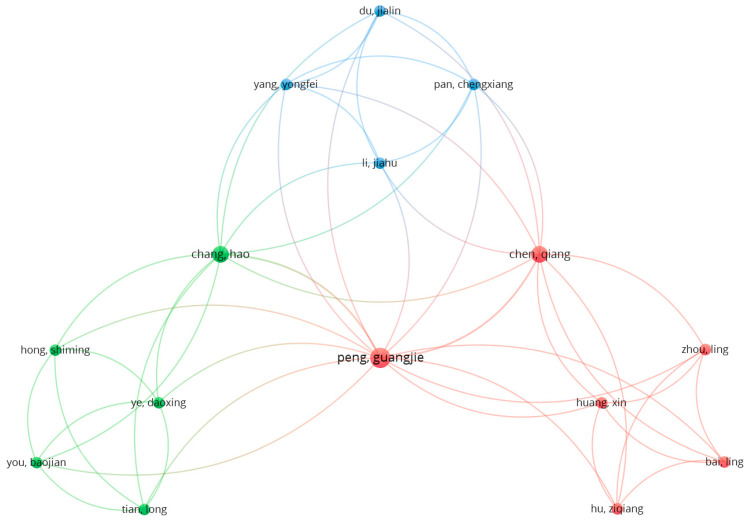
The largest set of connected items based on co-authorship links. Source: own development using VOSviewer software [72].

To supplement the conducted analysis, a co-occurrence of authors was investigated using VOSviewer software and Excel software. A total of 222 authors were identified from the selected papers. Figure 14 shows the results for the top 15 authors with the highest number of co-authorship connections. The author with the most links (16) is Guangjie Peng from China, who co-authored three papers [111,112,113]. Two other authors, Qiang Chen [111,112] and Hao Chang [112,113], also from China, each have a link strength of 11.

**Figure 15:**
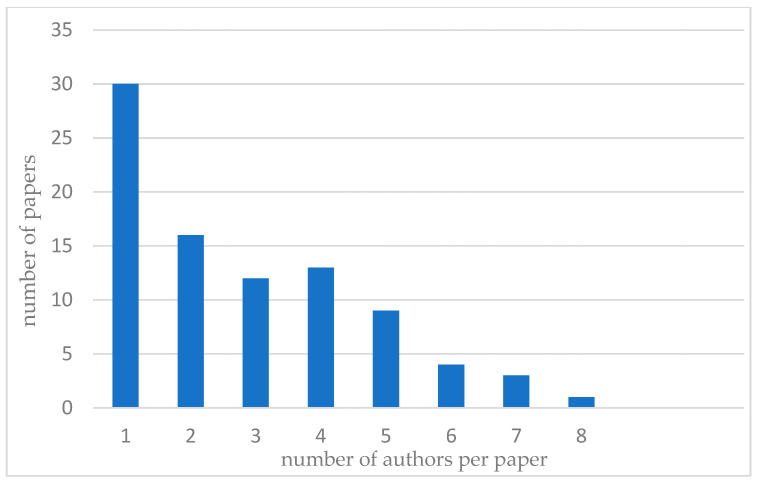
Distribution of publications per number of authors.

Furthermore, Figure 15 presents the distribution of publications based on the number of authors per paper. The findings reveal that single-author papers are the most common (30 publications). In contrast, papers with larger research teams (more than five authors) are less frequent in the analyzed sample.

**Figure 16:**
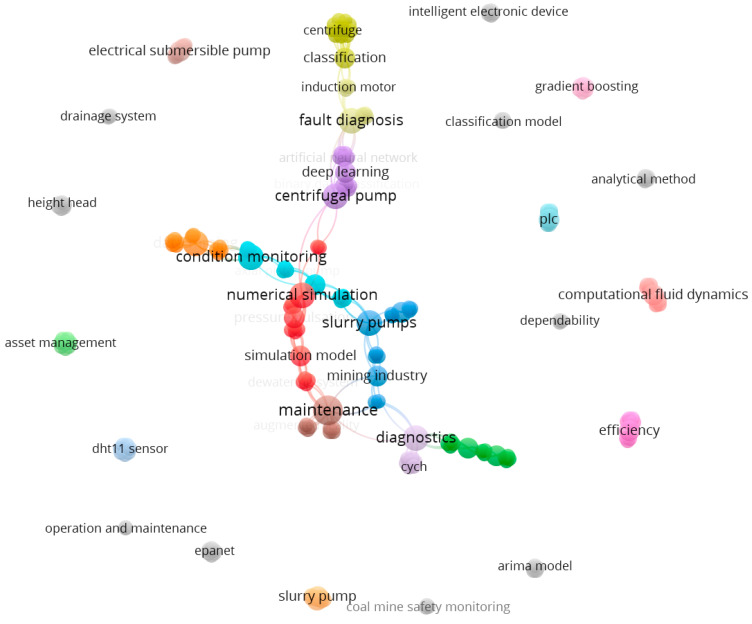
Mapping of the keywords that have occurred in the selected publications at least once. Source: own development using VOSviewer software [72].

The last part of the bibliometric analysis was a keyword co-occurrence analysis based on using VOSviewer software. The initial study focused on the keywords that occurred in the publications at least once. As a result, 220 keywords were identified for the selected papers (Figure 16). The most used words were maintenance (17 links), diagnostics (16 links), centrifugal pump (15 links), and fault diagnosis (15 links). The results present the used keywords in 28 clusters.

**Figure 17:**
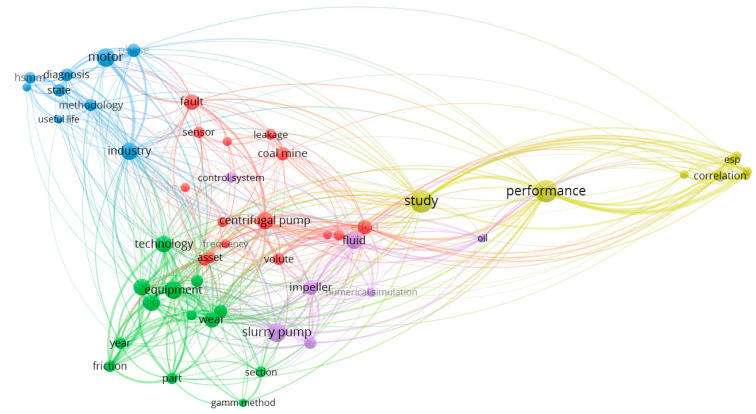
Mapping of the keywords with the largest occurrence. Source: own development using VOSviewer software [72].

A more detailed analysis was focused on developing a co-occurrence map based on the text from the titles and abstracts of the selected papers. The analysis focused on the 52 terms that have occurred at least five times in the papers (Figure 17). The obtained results are classified into five clusters.

The largest cluster (fifteen items, red one in Figure 17) is strongly associated with terms related to mining operations and machinery, such as centrifugal pump*,* coal mine, and open pit mine. This cluster represents a body of research focused on the operational performance, failure modes, and monitoring of mining environments, where equipment reliability and efficiency are critical.

The second cluster of publications (thirteen items, green one in Figure 16) reflects research focused on the technological aspects of equipment maintenance, emphasizing wear and friction in components such as pump parts.

The third cluster (nine items, blue one in Figure 17) is centered around assessing and extending equipment lifespan in industrial settings, particularly in the context of pump maintenance in the mining industry. This cluster likely represents research focused on developing and applying diagnostic methodologies to evaluate equipment conditions, predict failures, and optimize maintenance strategies.

The next cluster (eight items, yellow one in Figure 17) is strongly related to evaluating pump performance in real-world mining conditions, particularly for submersible pumps used in underground and deep-well applications. Indeed, this cluster represents research that combines experimental and field-based approaches to improve the understanding of submersible pump behavior, assess their performance in varying downhole conditions, and develop strategies for enhancing their operational lifespan in mining applications.

In the end, the fifth cluster (seven items, violet one in Figure 17) is closely related to the design, optimization, and performance analysis of pumps, particularly slurry pumps used in the mining industry. This cluster represents research focused on both theoretical and practical aspects of pump design, particularly for heavy-duty applications like slurry transport in mining. The studies within this group likely explore ways to enhance impeller efficiency, reduce wear, and implement advanced control strategies to improve the longevity and performance of pumps operating in harsh conditions.

The performed bibliometric analysis introduces the comprehensive content-based analysis, which is carried out in the next section.

### 4.2. Content-Based Analysis

As a result of the conducted research, following the methodology adopted (Figure 7), we focus on the content-based analysis.

**Figure 18:**
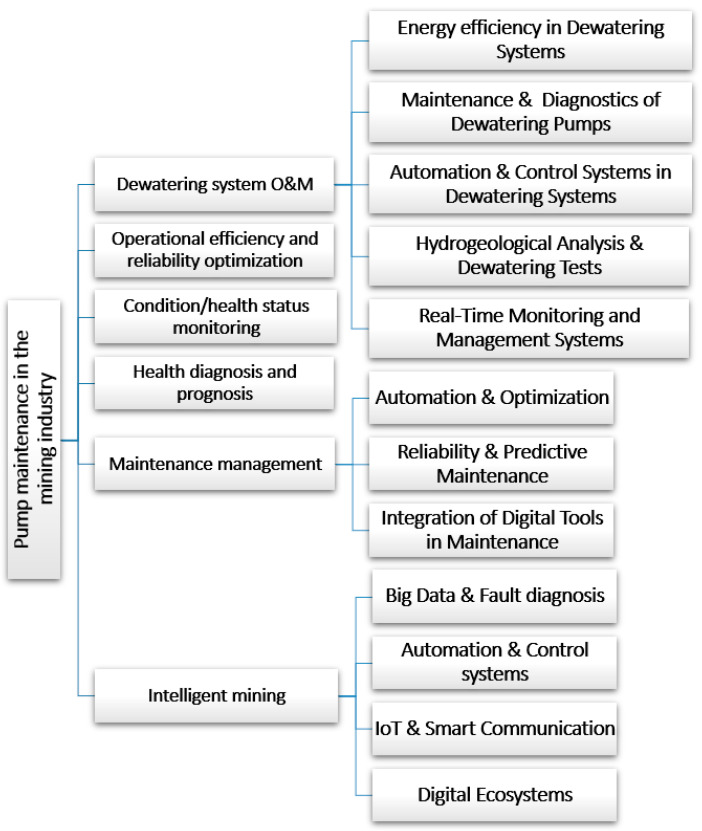
The main areas in the context of pump maintenance in the mining sector. Source: own contribution.

The identification of the main problems and issues raised in the context of pump maintenance in the mining industry was based on an extensive review of the available literature. The prepared literature analysis was also supplemented by review publications in the area of pump maintenance (e.g., works [34,74,76]). As a result of the research carried out, six core research areas were defined, which have been most extensively developed over the last twenty years (Figure 18). These are discussed in detail in the next subsections.

#### 4.2.1. Dewatering System Operation and Maintenance

The first research area under consideration is dewatering system operation and maintenance. According to the theory background presented in Section 2, dewatering systems play a critical role in the mining industry, particularly in operations where the removal of excess water from excavated areas or slurry is essential to maintaining operational efficiency and safety. Effective dewatering ensures that mining operations continue without interruptions caused by water accumulation, which can lead to flooding, equipment damage, and hazardous working conditions. These systems are primarily used to control water levels in open-pit and underground mines, manage water from tailings ponds, and handle the water content in slurry during processing.

The operation and maintenance of dewatering systems involve a variety of technologies and processes designed to remove water efficiently, whether it is groundwater, rainwater, or process water. Common methods include pumping, filtration, and evaporation, which rely on different types of pumps, drainage networks, and treatment processes. Maintaining the systems in peak working conditions is vital, given the critical nature of dewatering in preventing operational disruptions. Regular maintenance ensures that the pumps and other components perform optimally, reducing the risk of system failure and associated downtime, which can be costly in terms of both time and resources.

The twenty-three articles in this field provide a wide range of perspectives on dewatering system operation and maintenance, covering aspects such as system design, operational challenges, performance optimization, and the application of new technologies. These papers offer valuable insights into best practices for managing dewatering systems, including integrating automated monitoring systems, predictive maintenance strategies, and optimizing energy usage in pump operations. By improving the efficiency and reliability of dewatering systems, mining companies can enhance productivity, reduce environmental impact, and ensure safer working conditions for their personnel. In addition, the operation and maintenance of dewatering systems involve a variety of technologies and processes designed to remove water efficiently, whether it is groundwater, rainwater, or process water. Common methods include pumping, filtration, and evaporation, which rely on different types of pumps, drainage networks, and treatment processes. Maintaining the systems in peak working conditions is vital given the critical nature of dewatering in preventing operational disruptions. Regular maintenance ensures that the pumps and other components perform optimally, reducing the risk of system failure and associated downtime, which can be costly in terms of both time and resources.

One of the works in this area [114] offers a historical overview of dewatering pump technology, from steam-powered pumps in the 18th century to the electric pumps of the late 19th century. It highlights the evolution of pump technology and its importance in the mining industry’s dewatering systems. In another work [115], the authors discuss the safety issues related to damaged manholes in urban drainage systems. It presents a system for monitoring drainage conditions using sensors and the Internet of Things (IoT), which could provide useful insights for improving water management systems in mining sites, particularly in urban mining operations. Additionally, the paper [116] presents various dewatering equipment used in mining, including centrifugal pumps and filter presses. It focuses on energy-efficient, low-maintenance solutions, such as hybrid pumping systems and continuous steam filtration. The paper discusses the cost-effectiveness and performance improvements these technologies bring to mining dewatering processes. In addition, the technological advancements are discussed in [117,118]. The possibility of hose pump application in abrasive mining applications is presented in [119].

Another research area is connected with dewatering system reliability issues. In work [120], centrifugal pump connections are explored in series and parallel for dewatering processes in mining. The article investigates how these configurations affect pump efficiency, energy consumption, and reliability at different operational conditions (e.g., varying rotation speeds). The research concludes that the parallel connection is optimal for higher flow rates, while the series connection is suited for higher discharge heads. The problem of selecting a reliable dewatering pump for mining applications is investigated in [56,57] and continued in [121]. Another study [122] investigated the importance of pump availability in improving load-shifting performance in South African mining dewatering systems. The research introduces a methodology for investigating how reduced pump availability negatively impacts performance, highlighting significant cost increases related to pump unavailability. It emphasizes the critical link between operational efficiency and pump availability, especially in peak electricity demand periods. The next paper is focused on the dewatering pumps’ operational effectiveness [123]. This study focuses on the importance of effective operation and maintenance of dewatering pumps in underground mining, particularly the challenges posed by energy consumption and pump reliability. The study underscores the need for regular maintenance to minimize energy consumption and reduce operational costs. In addition, a mining automatic drainage monitoring and control system based on Siemens PLC (Siemens 314C-2DP) and KingView 6.53 software in the upper computer is presented. Energy efficiency and cost-effectiveness are discussed in [124]. This paper examines the energy efficiency of main dewatering pumps in mining operations, noting that excessive operational time between maintenance cycles can significantly increase energy consumption. It emphasizes the importance of monitoring and optimizing pump usage to reduce operational costs. The analysis was performed for the case of an underground mine in Poland. The energy efficiency problem in mining companies due to pumps and pipework operation is continued in, e.g., [125].

Energy consumption prediction is also under investigation by the author of [126]. The paper investigates machine learning models, such as random forest and k-nearest neighbors, to predict the energy consumption of underground dewatering pumps in gold mines. The results show that random forest outperforms other models, presenting an opportunity for predictive maintenance and energy optimization in mining operations. Another aspect of pump energy efficiency is investigated in [127]. This paper explores the impact of dewatering pump inefficiency in mining due to oversized pumps that are initially required for high pumping rates but often operate inefficiently during maintenance pumping. It discusses the importance of reviewing pump operation regularly to reduce electricity consumption, lower greenhouse gas emissions, and cut operating costs. The focus is on the lifecycle costs of pumps, with energy usage being the most significant cost. The results suggest that substantial savings could be achieved by replacing or optimizing pumps, especially considering future carbon taxes. The last work in the area of energy management is [58]. This paper discusses the monitoring and management of a dewatering system at the Drmno lignite open pit mine in Serbia. The system includes approximately 200 drainage wells with a total pump capacity of 2 MW, which removes 15 million cubic meters of water annually. The paper critiques the existing monitoring system and proposes a computer-assisted monitoring and management system to optimize dewatering processes, improve energy efficiency, and track operational costs.

Fault diagnosis for mining pumps in dewatering systems is the next topic under study. In work [128], a method combining minimum entropy deconvolution (MED), wavelet packet decomposition (WPD), and radial basis function neural networks (RBFNN) is proposed for diagnosing faults in mine drainage pumps. The approach effectively reduces noise interference and enhances fault recognition, boasting a high diagnostic accuracy of 94.44%.

The problem of mine dewatering systems monitoring and diagnostics continues to be addressed, e.g., in work [129]. This study highlights the importance of regular maintenance and monitoring of pumps in mine dewatering systems. It presents the preliminary results of diagnostic tests on pumps in a mine shaft dewatering system, using vibration analysis, thermal imaging, and acoustic testing to assess performance. The study emphasizes the need to choose the appropriate maintenance methods to ensure operational safety and reduce downtime, along with an initial operational test plan for further analysis. This problem is also investigated in [130]. This paper focuses on diagnosing the main pump drive systems in mines, with an emphasis on the supply, control, and drive systems. It describes the use of a PLC-based automatic pump control system, focusing on monitoring pump parameters and identifying failure conditions. The paper presents the results of diagnostic investigations, including real-time monitoring and visualization of pump performance in industrial plants.

The issues of cost-effective monitoring and control systems for underground drainage pumps in coal mines are investigated in works [131,132]. The authors present a solution in the first work utilizing a 32-bit ARM7 microcontroller and a touchscreen interface. It emphasizes the importance of user-friendly human-machine interfaces (HMIs) for efficient system control and monitoring. The system incorporates a graphical LCD to display pump parameters, which can be analyzed and adjusted based on real-time data [131]. The second paper outlines the design of a system based on Siemens S7-200 PLC and a Delta DOP-B touchscreen. The system monitors pump parameters, controls startup and shutdown actions, and gives water-level alarms when necessary. The system is designed to be reliable, cost-effective, and suitable for promotion in similar mining operations.

Another type of research problem being investigated recently regards hydrogeological conditions in coal mines. The authors in [133] present a three-step drawdown dewatering test to estimate water yields and hydrogeological conditions in coal mines, particularly focusing on the Siwan coal mine. The test combines steady and unsteady flow methods to assess aquifer storage and water yield predictions. The results offer insights into water availability and dewatering challenges, helping inform mine water management strategies.

In conclusion, the operation and maintenance of dewatering systems in mining operations involve a variety of technologies and processes aimed at efficiently removing water, such as centrifugal pumps, filtration, and evaporation. However, the literature presents contradictory views regarding the optimal selection of technologies. For instance, some studies advocate for low-maintenance solutions like hybrid pumping systems, as seen in [116], while others emphasize the costs associated with longer maintenance intervals [124]. Moreover, discussions on energy efficiency in dewatering systems show a lack of consensus on the best approaches. Papers like [124,127] stress the importance of optimizing pump operation to reduce energy consumption, but the results are not universally applicable, particularly in mines with specific energy needs. Predictive tools, such as those discussed in [126], highlight the potential for energy optimization through machine learning, but concerns about their reliability under unstable conditions persist. In terms of system reliability, research on pump configurations (e.g., [120,121]) suggests that parallel connections are better for higher flow rates, while series connections are more suitable for higher discharge heads. However, there is no clear guidance on when and where each configuration should be applied, leaving room for interpretation depending on the mine’s operational specifics. Furthermore, studies on pump monitoring and fault diagnosis (e.g., [128,129]) emphasize the use of technologies like vibration analysis and thermal imaging, but the effectiveness of these methods can vary depending on the operating conditions and the diagnostic equipment used. This lack of standardization across diagnostic approaches often leads to differing results. The application of advanced technologies, such as IoT and automated monitoring systems, is widely discussed, with works like [115,116] noting their potential to improve efficiency. However, the literature also indicates that these technologies may not be universally applicable, especially in regions with specific hydrogeological conditions, as noted in [133]. Overall, the literature review reveals a growing trend of incorporating modern technologies to enhance the performance of dewatering systems, but it also underscores the need for clearer, more unified guidelines for selecting appropriate technologies. This will help address the contradictions and inconsistencies found in existing research, ensuring more consistent and effective recommendations for practice.

#### 4.2.2. Operational Efficiency and Reliability Optimization

Mining operations rely heavily on pumping systems for dewatering, slurry transport, and process water management. The efficiency and reliability of these pumps directly impact operational costs, energy consumption, and overall mine productivity. However, harsh working conditions, abrasive materials, and variable operating demands pose significant challenges to pump performance and maintenance strategies.

To enhance operational efficiency and ensure long-term reliability, research in this field focuses on optimizing pump selection, control strategies, predictive maintenance techniques, and failure prevention methods. Various approaches, including computational fluid dynamics (CFD), machine learning, and experimental studies, have improved pump performance, reduced downtime, and extended service life.

**Figure 19:**
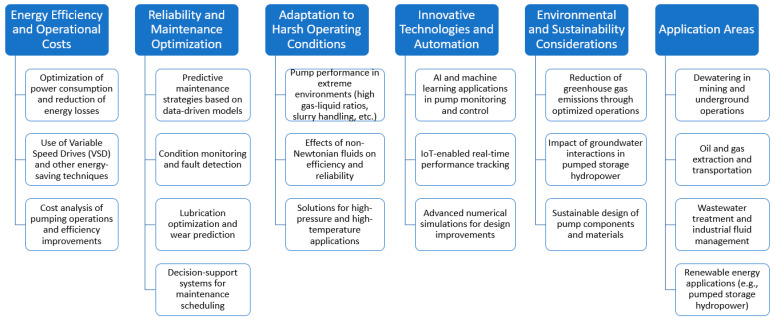
Main aspects of operational efficiency and reliability optimization in pump maintenance. Source: own contribution.

According to our study, we have identified 21 relevant studies that contribute to optimizing operational efficiency and reliability in mining pump maintenance. These works address key aspects such as energy consumption analysis, predictive maintenance using data-driven methods, impeller design improvements, and numerical simulations of pump behavior under different conditions. By synthesizing these research contributions, we aim to provide a comprehensive overview of current advancements and highlight future directions for optimizing mining pump performance. From analyzing the identified research works, we define six key research topics (Figure 19).

The first research aspect is focused on energy efficiency and operational cost optimization. Three identified studies [120,134,135] focus on optimizing pump operations to reduce energy consumption. Article [134] identifies inefficiencies in mine drainage systems. It examines the operating costs of electric pumps used in dewatering the Artois uranium mine. An energy balance was conducted over three years, revealing high specific energy consumption and inefficiencies in the current drainage system. The findings highlight areas for potential optimization. Next, ref. [135] develops an optimization model to enhance the scheduling process. Indeed, this research presents a modified EPANET2 toolkit (ETTAR) that enables rule-based control optimization for pump scheduling. The results demonstrate that optimizing pump control based on multiple conditions (e.g., tank levels and time of day) reduces operational costs while ensuring robust performance. The third article [120] explores centrifugal pump configurations, highlighting the advantages of parallel and serial connections in energy-efficient dewatering.

The second key research topic is focused on reliability and maintenance optimization issues. Research works [63,136,137] emphasize predictive maintenance strategies. The first work [63] applies data mining to predict lubricant service life in water injection pumps, ensuring reliability. Various algorithms, including gradient boosting and random forest, were tested, with results indicating that data-driven predictive models can effectively support pump reliability management. The authors in [136] explore flow characteristics in pump as turbine (PAT) systems, focusing on vortex formation, pressure pulsations, and rotor-stator interactions. The findings help improve PAT stability and efficiency by optimizing flow guidance and reducing pressure fluctuations. Later, in [137], a data-driven model integrating neural networks and Markov processes is proposed for optimizing pump scheduling in wastewater treatment plants. The model minimizes operational and maintenance costs through hierarchical particle swarm optimization, demonstrated in two case studies.

The third key research area is connected with adaptation to harsh operating conditions. Studies like [112,138,139] address pump performance in extreme conditions.

The first study [138] examines the impact of free gas presence on ESP performance under downhole conditions. Correlations predicting two-phase gas-liquid pump performance were developed using both field and laboratory data. The findings help optimize ESP operation but are recommended mainly for conditions similar to those in studied wells. The next article [139] focuses on handling non-Newtonian fluids in drilling applications. The study simulates the performance of centrifugal pumps transporting drilling fluids, showing that they exhibit lower head and efficiency but higher shaft power consumption than water pumps. It provides insights into flow patterns, pressure pulsations, and energy losses, contributing to pump design improvements. In another article [112], the authors introduce an impeller with splitter blades to reduce energy loss and wear in slurry pumps. By analyzing vortex structures and entropy generation, they demonstrate that the new design improves efficiency and durability, providing guidelines for pump optimization in mining and energy industries.

The next key research topic is connected with innovative technologies and automation introduction in order to improve pump operations. Article [140] evaluates LiDAR for mine inspections, demonstrating its feasibility for non-critical applications. The research assesses the accuracy and applicability of the iPhone 13 Pro LiDAR scanner for mining shaft inspections. Results indicate that while it lacks high precision, it is suitable for rapid inspections, offering a cost-effective alternative for preliminary 3D mapping. In addition, ref. [141] optimizes vacuum pump performance through adaptive discharge area control. This study examines how varying discharge areas impact liquid-ring vacuum pumps’ inspiratory capacity and power consumption. Experimental and numerical results confirm the importance of optimizing discharge areas to enhance compression efficiency and practical applications.

The last research aspect regards environmental and sustainability improvement in pump operations. In this area, ref. [134] highlights the high energy cost of mine drainage, emphasizing the need for efficiency improvements. Additionally, research work [142] explores UPSH as an alternative for renewable energy storage, balancing efficiency with environmental impact. In more depth, this numerical study evaluates the influence of groundwater interactions on UPSH system efficiency. The results show that groundwater exchanges enhance efficiency but also increase environmental impact, requiring a balance between energy performance and sustainability.

Additionally, we have identified another research area focused on implementation challenges and case studies related to mining pump maintenance. This category includes publications that examine real-world applications [57,119], practical deployment issues [143,144,145], and case-specific solutions for improving pump performance and reliability [146,147,148,149]. These studies provide valuable insights into the practical constraints, operational limitations, and industry best practices necessary to successfully implement advanced maintenance strategies in mining operations.

In conclusion, the reviewed literature offers various strategies for optimizing mining pump performance, focusing on energy efficiency, reliability, adaptability, and sustainability. However, differences in methodologies and results highlight the need for a more unified approach. Studies on energy efficiency and predictive maintenance, such as those by [63,134], show contrasting methods, suggesting that no single approach is universally applicable. Similarly, research on harsh conditions and pump design improvements, like [112,138], reveals inconsistencies in findings across different fluid types and pump configurations.

Innovative technologies, including [140] LiDAR for inspections and ref. [141] adaptive discharge control, show promise but face practical limitations. Environmental concerns raised by [134,142] emphasize the need to balance energy efficiency with sustainability.

Case studies, such as [57,119] or [146,147,148,149], offer valuable insights but lack broader applicability across diverse mining environments. While progress is evident, future research should focus on integrating these approaches and addressing existing contradictions to develop standardized, universally applicable solutions.

#### 4.2.3. Condition/Health Status Monitoring

Condition monitoring (CM) and health status monitoring (HSM) are essential techniques used to ensure the optimal operation and longevity of pumps in the mining industry. These methodologies involve continuously assessing the condition of equipment—such as pumps—by collecting and analyzing real-time data to detect any deviations or signs of wear that may lead to failure. The goal is identifying potential issues before they lead to significant breakdowns or inefficiencies, minimizing unplanned downtime and optimizing maintenance schedules.

The thirteen selected articles on condition/health status monitoring in mining pumps focus on various aspects of monitoring pump performance and detecting potential faults that may affect their operation. These studies employ diverse methodologies, technologies, and models to analyze the health of pumps and related systems. The first article [150] proposes a model for monitoring the leakage and diffusion of gas drainage pipelines in underground coal mines. The use of field data to validate the model ensures that gas sensors downstream of leakage points can effectively detect gas leaks. While not focused directly on pumps, it contributes to the broader context of safety and equipment health, highlighting the importance of monitoring systems in mining operations. It shows how a real-time monitoring system can help prevent accidents related to gas leaks.

The second work [53] focuses on the problem of wear monitoring in slurry pumps. This study investigates the wear status of slurry pumps using unsupervised clustering ensemble methods to assess and measure the wear of wetted components, like impellers. The article emphasizes the importance of condition-based monitoring to identify pump wear before it results in unplanned outages, showcasing how predictive techniques can optimize maintenance schedules and prevent costly downtime.

The next four works are also dedicated to the problems of slurry pump wear analyses [111,113,151,152]. In the first work [111], the authors focus on the wear patterns of slurry pump impellers, utilizing a multiphase flow model, and investigate how operational conditions such as flow rate and particle concentration affect wear. The findings suggest that wear-prone areas can be identified, which helps improve the design and maintenance strategies for slurry pumps, thus reducing unplanned downtimes and enhancing pump efficiency. Similar to this study, ref. [113] analyzes the wear characteristics of slurry pumps under varying operational conditions. The study emphasizes how different particle sizes and concentrations contribute to the wear of impellers. These insights can be used to improve slurry pump designs and monitoring systems to optimize their performance and lifespan. The work [151] discusses the main challenges and future pump-related technologies in relation to their possible development. The last work [152] presents Metso’s plant control monitoring software with an example of its use in slurry pump operation.

The next work [153] introduces a model to predict abnormal ESP operations when the pump is compromised by free gas. The research provides a method for monitoring ESP health and anticipating failures using nodal analysis and field case studies. The detection of abnormal operation parameters allows for early interventions to prevent premature pump failure and production losses.

Intelligent electronic devices (IEDs) are under investigation in another work [154]. The paper discusses the application of IEDs in monitoring motor control centers and drives used in mining operations. By using the IEC 61850 standard, these devices can detect potential faults and allow for real-time monitoring of motor health. This highlights the importance of adopting intelligent systems for preventive maintenance in mining machinery.

In another work [155], the authors investigate hydraulic transmission system health monitoring. In this study, the health of hydraulic transmission systems, including axial piston pumps, is monitored by analyzing leakage flow characteristics. The simulation-based approach focuses on detecting performance degradation due to increased leakage and wear, which indicates pump health. This is a preventive measure aimed at minimizing system failures and improving reliability.

The problem of axial piston pump condition monitoring is later continued in work [156]. This article develops a mathematical model for axial piston pumps, which uses pressure and flow measurements to identify changes in pump health, particularly related to wear and leakage. The research emphasizes pressure control’s significance in maintaining optimal performance and preventing system breakdowns. This approach underlines the importance of continuously monitoring operational parameters for early fault detection.

The next work is worth investigating [157], focusing on centrifugal pump health monitoring using fluid pressure. This study focuses on detecting faults like blockages and cavitation in centrifugal pumps by analyzing fluid pressure signatures. The deep learning-based binary classification method allows for the identification of fault types and severity, offering a robust way to monitor pump health and take timely corrective actions.

The next work [158] presents a cost-effective solution for condition monitoring by using a central power measurement device to analyze the power consumption patterns of industrial devices, including pumps. Applying spectral analysis and data mining models demonstrates how monitoring power usage can be a predictive tool for identifying real-time equipment failures.

Safety issues and real-time monitoring in coal mining are investigated in work [159]. The article highlights the use of multi-sensor systems to monitor various equipment, including pumps, in coal mining operations. By collecting and analyzing data from sensors that monitor gas concentration, temperature, and pressure, this system provides insights into the health status of the equipment and ensures the safety of mining operations.

**Figure 20:**
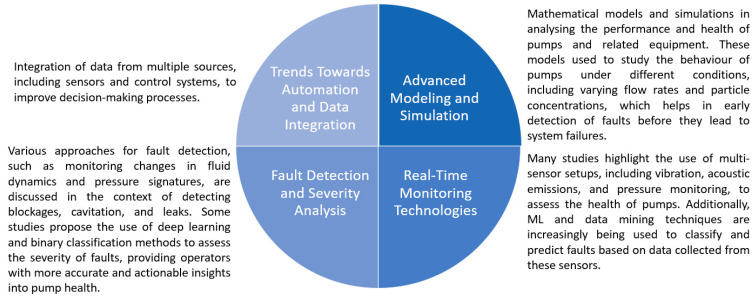
Main aspects of condition/health status monitoring in pump maintenance. Source: own contribution.

In summary, the articles on condition/health status monitoring in pump maintenance highlight several key insights and future trends. The research focuses on advanced modeling, simulation, and real-time monitoring technologies used to predict failures, improve maintenance strategies, and ensure operational safety (Figure 20).

In conclusion, the reviewed literature on condition and health status monitoring (CM/HSM) in mining pumps provides valuable insights into optimizing pump performance and preventing failures. While the studies contribute to understanding pump health using various methods, such as wear monitoring, simulation-based analysis, and real-time data collection, their approaches have notable differences. Some studies, like [53,111], highlight the use of predictive models to monitor wear and operational conditions, which can effectively prevent unplanned downtimes. However, methodologies differ, with some focusing on specific pump types or operating conditions, raising concerns about their findings’ generalizability across different mining environments.

For instance, the application of intelligent electronic devices (IEDs) [154] for real-time monitoring contrasts with other predictive maintenance techniques, showing diverse approaches in addressing similar issues of fault detection. Similarly, studies on slurry pump wear [111,113] and ESP operation [153] point to the importance of condition-based monitoring, yet their results sometimes conflict with the effectiveness of specific sensor-based models. Moreover, while several studies emphasize real-time monitoring, such as [159], others focus on simulation-based approaches, like [156], which may not always reflect actual operating conditions.

Future research should aim to integrate these varied approaches into more unified systems, balancing automation, predictive maintenance, and real-time data collection. Addressing contradictions in methodologies and results will be crucial for developing more reliable, broadly applicable monitoring systems for mining pumps.

#### 4.2.4. Health Diagnosis and Prognosis

In the context of mining pump maintenance, health diagnosis and prognosis play crucial roles in ensuring the continuous and efficient operation of pumps, which are integral to various mining processes such as dewatering, slurry transport, and fluid handling. Health diagnosis refers to the process of detecting, identifying, and assessing the current condition or “health” of the pump and its components. This can involve identifying faults, wear, and degradation in real time, often using sensors, condition monitoring systems, and diagnostic algorithms.

On the other hand, prognosis is the prediction of the future health of the pump, particularly in terms of its remaining useful life (RUL) and potential failures. Prognostic models utilize data from diagnostic tools, historical performance, and environmental conditions to forecast when a pump might fail or require maintenance. These predictions allow for proactive measures to be taken before failures occur, preventing costly downtimes and unscheduled maintenance.

Research in this area is advancing with the use of data-driven approaches, such as machine learning, predictive analytics, and condition-based monitoring systems. These technologies help enhance the accuracy of diagnostic assessments and prognostic predictions. Furthermore, this research field is becoming increasingly important as mining operations strive for higher levels of automation and cost-effective operations, where predictive maintenance models are becoming indispensable tools for ensuring continuous production without sacrificing equipment reliability.

By focusing on health diagnosis and prognosis, mining pump maintenance strategies can move from traditional, reactive methods to a more proactive and predictive approach, ultimately optimizing the performance and reliability of pumps in harsh mining environments. This research area is vital for improving the overall sustainability and efficiency of the mining industry, contributing to smarter, data-driven decision-making processes in pump management.

**Figure 21:**
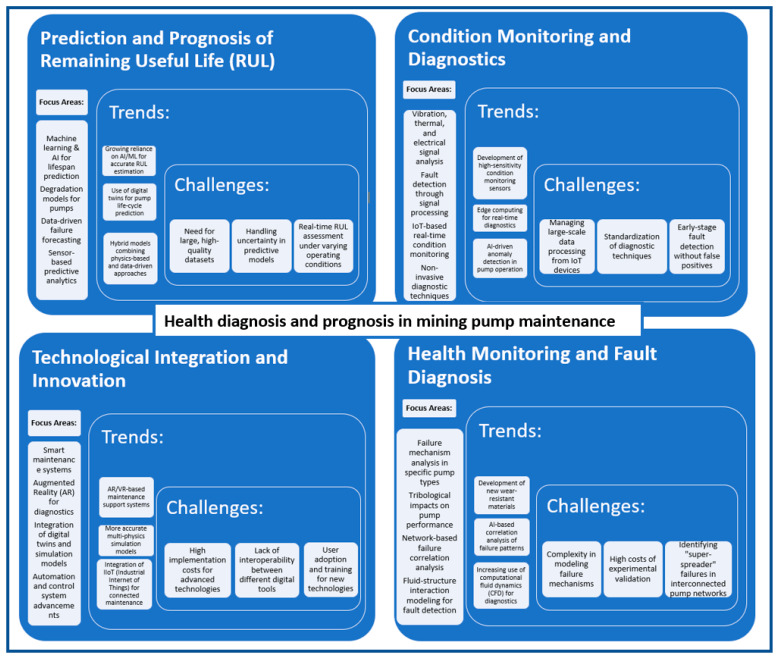
Main aspects of health diagnosis and prognosis in mining pump maintenance. Source: own contribution.

In our study of this research area, we have identified 21 selected articles that are highly relevant and contribute to the advancement of health diagnosis and prognosis models in mining pump maintenance. These works focus on innovative methods, technologies, and case studies that improve diagnostic accuracy and prediction capabilities, further enhancing maintenance strategies and operational sustainability. The selected research works can be classified into four main research areas (Figure 21).

The first group focuses on methods and techniques for predicting the remaining useful life (RUL) of machinery and equipment, which is a crucial aspect of condition-based maintenance (CBM). Predicting RUL is vital for determining the optimal time for maintenance actions, preventing unexpected failures, and extending the operational life of systems. The techniques often involve advanced data analytics, machine learning algorithms, and statistical models to estimate how long a system or component will continue to function before failure.

Few papers focus on prediction models and algorithms by discussing the application of sophisticated models like segmental hidden semi-Markov models (HSMMs) and relevance vector machines (RVMs) to predict RUL. These models are built to handle the complexities and temporal patterns in real-world machinery operations, particularly in hydraulic and industrial pumps. One paper [160] presents an HSMM-based methodology for both diagnosis and prognosis in a unified framework capable of predicting the RUL of hydraulic pumps. It improves the recognition of pump states and helps predict remaining useful life (RUL), with testing showing improved recognition rates and prognosis capabilities over traditional models. Another paper on this topic [161] highlights the use of RVMs to predict the RUL of pump impellers, comparing their effectiveness to traditional exponential fitting methods. The model outperforms traditional methods by predicting RUL more accurately, demonstrating its effectiveness with field data. In addition, the methodologies are often applied to real-world scenarios to test their practical utility. The health monitoring of hydraulic pumps and oil sand pump impellers is used as a case study, where real operational data are analyzed to validate the models’ accuracy in predicting RUL.

The next work [162] presents a framework for multi-sensor-based health monitoring and prognosis of equipment using an adaptive hidden semi-Markov model (AHSMM). AHSMMs enhance the traditional hidden semi-Markov models (HSMM) by addressing computational challenges, making them more efficient for large-scale applications. The approach integrates sensor data to predict RUL accurately. This model is applied to monitor hydraulic pumps from Caterpillar Inc., demonstrating its effectiveness in real-world conditions. The study concludes that this method provides valuable insights into the degradation process and offers improved prediction accuracy compared to conventional models.

Another problem is defined in [163], where the authors use the auto-regressive integrated moving average (ARIMA) model to forecast the RUL of axial piston pumps used in hydraulic systems. The ARIMA model is especially useful for time-series forecasting, where it analyzes historical data, including parameters like leakage volume, to predict future performance. The study focuses on finding the optimal ARIMA model configuration, with the ARIMA (1, 1, 2) model showing the lowest forecasting error. The predicted RUL of the pump is 28 months, providing a solid foundation for maintenance planning and failure prevention in applications like mining and agriculture.

The application of relevance vector machines for predicting the RUL of slurry pump impellers in the oil-mining industry is presented in [54]. The study introduces a novel feature extraction process to tackle the non-stationary nature of slurry pump data, which is often characterized by fluctuating operational conditions. The RVM model, combined with a two-summed exponential function, successfully captures the degradation trend of the impellers, enabling the prediction of their RUL and helping avoid unplanned breakdowns. The model’s accuracy in predicting pump degradation is validated with real-world field data.

The last work in this area is [52]. This work explores the use of support vector machines (SVMs) to estimate the RUL of slurry pumps, which are critical in industries such as mining, waste treatment, and oil sands. Given the harsh, abrasive operating conditions, slurry pumps experience continuous wear, especially on components like impellers. Traditional maintenance strategies often fail to anticipate failures in time, leading to unnecessary part replacements or unplanned breakdowns. The study proposes a data-driven technique using SVMs to analyze the pumps’ sensor data, identifying the components’ health status and providing a reliable estimate of the RUL. The effectiveness of SVM is demonstrated through sensor data analysis, emphasizing the importance of sensor placement and frequency band selection for optimal results.

The second research field focuses on condition monitoring and diagnostics for continuously assessing the health of industrial machinery and systems in order to identify potential faults and prevent unexpected breakdowns. The main goal is to improve maintenance practices by enabling proactive actions based on real-time performance data, which helps in extending the lifespan of equipment, improving reliability, and minimizing unplanned downtime. The first work in this area [164] focuses on vibration-based monitoring for detecting faults in reciprocating compressors used on an oil rig in the Baltic Sea. Due to the strong impacts and disturbances in the vibration signals, the system must handle significant signal distortion. The paper introduces the use of enhanced resolution envelope analysis to address the challenges posed by these strong vibrations. The methodology is applied specifically to detect bearing faults in the compressor, highlighting the importance of selecting the appropriate diagnostic method based on the nature of the fault and the machinery’s operating conditions. This paper demonstrates the role of vibration analysis in machinery diagnostics, especially in harsh operating environments like offshore oil rigs.

Thermal imaging for fault diagnosis in three-phase induction motors is introduced in [165]. The study presents a fault diagnostic technique that analyzes thermal images to identify issues such as broken bars or faulty rings in the motor’s squirrel-cage rotor. The MoASoID (method of areas selection of image differences) technique is used to extract significant features from thermal images, which are then classified using machine learning techniques like nearest neighbor (NN), k-means, and backpropagation neural network (BNN). This approach provides a non-invasive, efficient method for identifying faults, thereby protecting motors from damage that could lead to significant downtime or economic loss.

Automated pump diagnostics using PLC control is investigated in the next work [130]. This work introduces an automated diagnostics system for pump drive systems, focusing on the supply system, control system, and drive part of the pumps. The diagnostics are integrated with a PLC controller, enabling real-time monitoring of various parameters to detect failure states. The paper discusses the effectiveness of the diagnostic system in industrial plants, where it was successfully verified. The diagnostic method helps in identifying operational issues in the pump system, such as power loss or mechanical failure, by continuously tracking and visualizing key parameters. This system supports online diagnostics and can be used for preventive maintenance, ensuring that pumps continue operating efficiently.

The last work [166] focuses on slurry pumps used in oil sand pumping operations, where the impellers are subject to continuous wear and erosion due to the abrasive nature of the liquid-solid mixtures they pump. The paper introduces two wear degradation indices—the moving-average mean and moving-average deviation wear indices—to track the health of the impellers over time. These indices are based on vibration signals collected from the pump, and their effectiveness is validated through real-world data. By assessing the degradation of impeller performance, this approach helps predict impeller failure and improve the reliability of slurry pumps, reducing costly breakdowns and extending their operational lifespan.

The third research field—technical integration and innovation—focuses on the incorporation of advanced technologies to enhance the efficiency, reliability, and predictive capabilities of industrial systems. This includes leveraging sensor-based diagnostics, dynamic modeling, augmented reality (AR), and machine learning algorithms to improve maintenance strategies and optimize system performance. In this area, ref. [167] proposes a novel approach to pump diagnostics that involves using the motor itself as a sensor, analyzing current and voltage signals to detect faults such as cavitation, hydraulic blockage, and dry running. This extends traditional motor diagnostics techniques to assess pump health, offering a more integrated and cost-effective solution for predictive maintenance. The research focuses on testing different classification methods to determine the most effective diagnostic approach. The next work [153] proposes advanced nodal analysis software (e.g., Schlumberger PIPESIM) that is used to monitor and predict failures in electric submersible pumps (ESPs) operating under high gas conditions. By analyzing pump intake pressure (PIP) and pump discharge pressure (Pdis), abnormalities in ESPs related to gas lock are diagnosed.

The oil and gas sector, especially in remote and extreme environments (e.g., Arctic regions), requires efficient maintenance strategies. As a result, in work [168], dynamic modeling is integrated with augmented reality (AR) (via Microsoft HoloLens 2) to enhance diagnostics and reduce maintenance time. AR allows technicians to access real-time system data, interactive maintenance guides, and predictive analytics, streamlining repair and servicing processes. The combination of AR with simulation-based diagnostics represents a breakthrough in remote maintenance assistance and digital twin applications.

The next work in this research field is focused on data mining and AI for predictive maintenance in centrifugal pumps [169]. This work applies big data analytics and machine learning (ML) techniques to process vast amounts of data generated during pump operations. The C5.0 Decision Tree (DeT) algorithm analyzes vibration signals (horizontal, vertical, and axial planes) and classifies pump health status.

The last work focuses on the application of fault diagnosis and fault tolerance in the areas of electrical, mechanical, and chemical engineering as well as computer science [170].

The next research field is focused on health monitoring and fault diagnosis in specific pump types. Health monitoring and fault diagnosis of pumps are critical for ensuring reliability and minimizing operational costs, particularly in industries like mining, power generation, and heavy manufacturing. The reviewed articles provide insights into various failure mechanisms, diagnostic methods, and predictive maintenance strategies across different pump types. [161] focuses on hydraulic plunger pumps used in coal mines. It challenges prior assumptions about suction valve failure, revealing that rotational wear and material loss, rather than impact wear, are the main causes of degradation. Using computational fluid dynamics (CFD) and nonlinear finite element analysis, researchers identified uneven flow fields and low-speed rotation as key contributors to premature wear. Improved valve materials based on these findings led to increased durability in field tests. The next study [171] presents a global analysis of energy consumption due to friction and wear in mining operations, where pumps play a significant role. The study found that 40% of energy in mineral mining is used to overcome friction, and additional energy is expended on manufacturing replacement parts. By implementing advanced materials, coatings, and lubrication technologies, energy losses could be reduced by up to 30% over 20 years, resulting in major economic and environmental benefits. In another work [172], the authors highlight a failure rate monitoring initiative for large industrial pumps in the power and mining sectors. Over four years, data were collected on 17 different pump types across multiple locations in the Czech Republic. The study presents baseline failure rate evaluations and identifies unexpected fluctuations in failure rates, providing potential solutions to improve pump reliability. The next work [173] explores cascading failures in pump networks, where failures in one pump can trigger additional failures in connected assets. Using complex network analysis on data from 5655 pumps and 50,000 work orders in a mining company, researchers identified “super-spreader” pumps whose malfunctions led to increased maintenance demands. The study suggests that preventive maintenance measures can be implemented to mitigate cascading effects by recognizing these patterns. The last paper in this research field [174] investigates piston diaphragm pumps used for transporting abrasive and aggressive slurries in high-pressure applications. The study proposes a fluid-structure interaction (FSI) simulation to analyze pressure pulsation behavior, allowing for more accurate predictions of deformation and fluctuations. This model helps optimize pump design and improve operational stability.

In the field of pump health diagnosis and prognosis, various methods and approaches have been proposed by researchers. These methods often differ in terms of the underlying technology, accuracy, computational requirements, and application in real-world systems. A critical analysis and comparison of these methods reveal both strengths and weaknesses, as well as contradictions in the findings of different authors. To facilitate a clearer understanding, the following Table 2 summarizes several key approaches, highlighting their respective advantages and disadvantages and the inconsistencies found in the conclusions across studies.

The comparison of different pump condition monitoring and RUL prediction methods reveals a range of strengths and limitations. HSMM offers high accuracy, but its complex computational requirements make it challenging for large systems, and its performance may degrade in unstable conditions. RVM, while accurate with lower data needs, is sensitive to data errors, leading to inconsistent results. ARIMA is simple and effective for time series data but struggles with irregular data and varying pump systems, showing inconsistent results across cases.

Vibration-based monitoring is cost-effective and provides early fault detection but may miss certain failure types and is affected by environmental noise. Augmented reality is useful for real-time diagnostics but comes with high costs and infrastructure demands, limiting its applicability. SVM is effective for sensor data analysis but requires large datasets and precise tuning and performs poorly with low-frequency measurements.

In conclusion, while these methods each have their merits, they also exhibit contradictions and limitations, underlining the need for further refinement and possibly integrating approaches to enhance their reliability in diverse real-world conditions.

#### 4.2.5. Maintenance Management

Maintenance management in the mining industry, particularly for pump systems, refers to the systematic planning, monitoring, and optimization of maintenance activities to ensure the reliable, efficient, and cost-effective operation of pumps used in water management, slurry transport, and hydraulic systems. It integrates preventive, predictive, and condition-based maintenance strategies to minimize unplanned failures, optimize energy consumption, and extend equipment lifespan [34].

Following this, we have identified six main research papers in this research area. The reviewed articles focus on various aspects of pump maintenance in mining operations, emphasizing automation, data-driven decision-making, and advanced maintenance strategies. Their central theme revolves around improving efficiency, reliability, and cost-effectiveness in mining pump systems through different technological approaches.

One key aspect explored in the articles is the automation of water pumping systems. The first article [175] discusses the importance of automating underground clear water pump stations to optimize energy consumption, prevent flooding, and reduce operational costs. Similarly, another article [176] presents a control method for pump speed regulation using a high-voltage synchronous electric motor drive and frequency converters, ensuring efficient water supply management. These approaches highlight the role of automation and real-time monitoring in optimizing pump performance.

Another significant theme in the revised area is data-driven maintenance and failure analysis. The GAMM method, discussed in two of the articles, provides a graphical analysis of equipment reliability and identifies anomalies affecting pump operations. By analyzing historical data, GAMM enhances decision-making in maintenance management and helps detect failures stemming from operational misuse, design flaws, or maintenance inefficiencies. This aligns with broader trends in predictive maintenance, where statistical modeling and reliability estimation are used to improve equipment longevity [177,178].

The role of computer-aided maintenance management is also highlighted in the discussion of computerized maintenance management systems (CMMS) applied to the Polish copper mining industry [179]. By leveraging CMMS data, mining companies can analyze failure intensities of critical components, such as hydraulic pumps in haul trucks, and refine their maintenance strategies accordingly. This case study emphasizes the shift from time-based maintenance (MTTR, MTTF, MTBF) to data-driven, condition-based maintenance, improving efficiency and reducing unplanned downtimes.

The last identified research work [180] presents a case study that discusses the successful implementation of a total asset management plan by Weir Minerals at the Tronox mineral sands mine in Cooljarloo, Western Australia. The key objectives of the asset management plan include enhancing asset reliability, minimizing maintenance costs, and optimizing operational efficiency. By leveraging Weir’s expertise, Tronox benefits from a structured maintenance strategy, reducing unplanned downtime and ensuring the longevity of critical equipment. This case study demonstrates how comprehensive asset management, strategic maintenance practices, and innovative equipment upgrades contribute to sustainable and cost-efficient mining operations.

**Figure 22:**
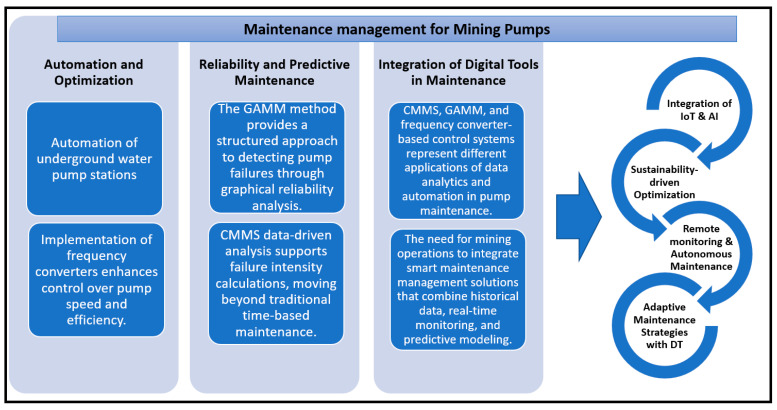
Main aspects of maintenance management for mining pump maintenance. Source: own contribution.

To sum up, effective maintenance management for mining pumps is essential for ensuring operational reliability, cost efficiency, and sustainability in mining operations. The six analyzed articles highlight different approaches to pump maintenance, emphasizing the role of automation, data-driven decision-making, and advanced control systems. The key insights from these case studies provide a comprehensive perspective on optimizing maintenance strategies to enhance pump performance and reduce operational risks. The six research works collectively demonstrate that effective pump maintenance management in mining requires a combination of automation, data-driven decision-making, and asset lifecycle optimization. Future advancements in AI, IoT, and sustainability-focused technologies will further revolutionize maintenance strategies, ensuring more efficient, cost-effective, and environmentally friendly mining operations (Figure 22).

#### 4.2.6. Intelligent Mining

In this research area, the authors focus on the aspects of intelligent mining. Intelligent mining refers to integrating advanced digital technologies to optimize and automate mining operations, enhancing efficiency, safety, and sustainability. It combines big data analytics, artificial intelligence (AI), the Internet of Things (IoT), cloud computing, automation, robotics, and digital twin technology to enable real-time monitoring, predictive maintenance, and autonomous decision-making [181,182].

A key aspect of intelligent mining is data-driven decision-making, where IoT sensors and AI algorithms continuously collect and analyze operational data. This enables predictive maintenance strategies that minimize downtime and extend equipment lifespan. Automation plays a crucial role by deploying autonomous vehicles, drilling systems, and robotic machinery, reducing the need for human intervention in hazardous environments. Additionally, intelligent mining leverages remote monitoring and control, allowing centralized operation centers to oversee and optimize mining processes in real-time.

The transition from reactive maintenance to predictive and proactive strategies is a defining feature of intelligent mining. Instead of responding to equipment failures after they happen, mining companies can now anticipate and prevent issues, leading to more resilient and cost-effective operations. Moreover, by integrating digital ecosystems, different mining components—such as pumps, conveyors, drilling equipment, and ventilation systems—can communicate and adapt dynamically, improving overall mine productivity.

In the context of pump maintenance, intelligent mining transforms traditional approaches by introducing real-time condition monitoring, AI-based fault detection, and automated control systems. IoT-enabled sensors track pump performance, while AI-driven models predict potential failures before they occur, reducing unplanned downtime. Digital twin technology further enhances maintenance strategies by simulating pump behavior under different conditions, allowing for optimized operational planning. These advancements ensure higher reliability, reduced maintenance costs, and improved operational efficiency [183,184].

As a result, in the defined research area, the authors identified four main research works that focus on intelligent mining in the context of pump maintenance. These articles focus on different aspects of intelligent pump system management in the mining industry, emphasizing the use of advanced technologies such as big data analysis, automation, adaptive control, and digital ecosystems. The common goal across all papers is to improve the reliability and efficiency of pump systems through intelligent monitoring, fault diagnosis, and operational optimization.

In the first article [185], the authors discuss the application of big data analysis in pump fault diagnosis. By leveraging machine learning and data-driven models, the fault diagnosis process becomes more intelligent, autonomous, and accurate. The study highlights how big data enable self-learning fault identification, reducing downtime and improving predictive maintenance strategies.

In another work [66], the development of an intelligent automated water intake system for open-pit mines, designed using LabVIEW and Siemens 1200 PLCs, is presented. The system integrates electric gate valves, ball valves, and various sensors to enhance operational efficiency. The software enables real-time monitoring, automated pump rotation control, and fault detection, reducing manual labor and increasing the system’s reliability.

A different approach is given in [186]. This article explores the implementation of frequency conversion constant pressure control systems for emulsion pump stations in mining operations. The research integrates IoT, 3D laser scanning, and 5G communication to enhance automation, fault self-diagnosis, and remote monitoring. By analyzing the friction coefficients of different drawing fluids, the study emphasizes how environmental factors influence the selection of pump control strategies.

The fourth article [187] discusses the concept of a digital ecosystem in mining, where networks of sensors, pumps, and control systems interact dynamically. The paper highlights the scalability and adaptability of digital ecosystems, from monitoring a localized pump system to coordinating multiple mining operations worldwide. Such integration enhances real-time decision-making, predictive maintenance, and operational efficiency.

**Figure 23:**
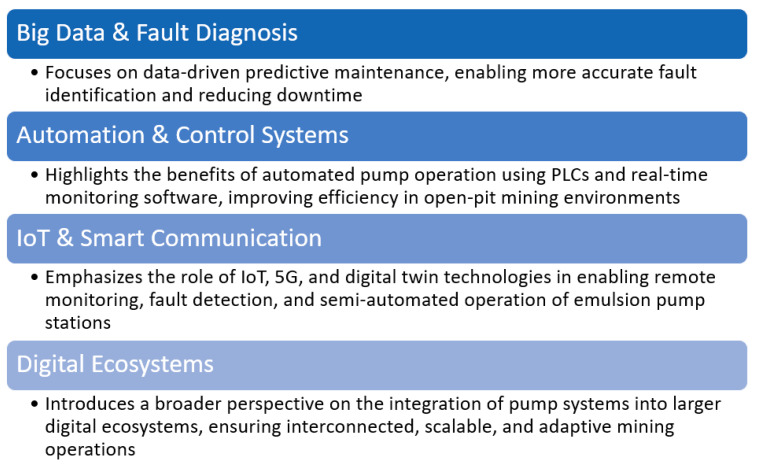
Main aspects of intelligent mining in the context of pump maintenance. Source: own contribution.

All four papers contribute to the broader theme of intelligent mining and pump system maintenance, but they differ in their technological focus and approach (Figure 23).

The evolution of pump maintenance in mining reflects a broader shift towards intelligent mining operations, where real-time data analysis, automation, and digital interconnectivity play a central role. These papers collectively illustrate how the industry is moving from reactive maintenance towards proactive and predictive approaches, ultimately leading to increased efficiency, reliability, and sustainability in mining operations. Based on their analysis, we may state that the evolution of intelligent mining reflects a clear shift from traditional, reactive maintenance approaches to fully automated, data-driven, and predictive maintenance strategies. This transformation is driven by the integration of big data analytics, IoT, AI, and digital twin technology, allowing mining operations to move from manual fault detection toward real-time monitoring, automated diagnostics, and remote control. Adopting these technologies ensures higher operational efficiency, improved equipment longevity, and reduced downtime.

A crucial element of this transition is the integration of IoT and smart communication technologies, such as 5G-enabled remote monitoring and cloud-based data management, which enable centralized control and seamless coordination between different mining components. This interconnectivity enhances operational scalability, allowing companies to optimize their mining infrastructure while reducing labor-intensive processes. Additionally, the scalability of digital ecosystems plays a vital role as mining companies increasingly connect localized pump systems to global networks, ensuring synchronized operations and predictive decision-making across multiple sites.

Ultimately, in the mining industry, the evolution of maintenance strategies has been increasingly influenced by intelligent mining technologies, which aim to optimize efficiency, reduce downtime, and enhance the sustainability of operations. Intelligent mining integrates advanced technologies like big data analytics, Internet of Things (IoT), artificial intelligence (AI), digital twins, and automation to support real-time monitoring, predictive maintenance, and autonomous decision-making. The research on intelligent pump system management in mining explores various technological approaches to enhance the reliability and efficiency of pump maintenance. Below, in Table 3, we summarize and critically analyze the main approaches presented in the literature, comparing their strengths, weaknesses, and practical implications.

The presented table summarizes various technological approaches to intelligent mining in the context of pump maintenance, illustrating the benefits and limitations of each. Approach 1, based on big data and machine learning, provides accurate fault detection and autonomous diagnostics, but it requires high-quality data and faces integration challenges with older systems. Approach 2 utilizes IoT and 5G technology for remote monitoring, offering the advantage of real-time control and reduced operational costs. However, the initial costs and integration with legacy systems may pose challenges. Approach 3, focusing on automation and digital twin technology, allows for operational optimization and predictive maintenance. While it offers high efficiency, it demands significant investments in infrastructure and may not be easily implemented in older systems. Approach 4, relying on PLC and automation, reduces manual labor and is easy to integrate with existing systems. However, it has limited scalability in large installations and depends on the specific PLC infrastructure in place.

The comparisons show that while all approaches aim to improve efficiency and reduce downtime, the choice of technology must align with the specific operational context of the mining company. Larger and more technologically advanced operations may benefit from integrating multiple technologies, such as combining IoT, AI, and digital twin technology. However, these approaches require substantial investments and may not be feasible for all mining operations.

In conclusion, the evolution of intelligent mining reflects a shift from reactive to proactive and predictive maintenance strategies. By integrating automation, real-time data analysis, and digital connectivity, mining operations can move toward more resilient, cost-effective, and sustainable practices. Future advancements will likely focus on further improving the scalability and interoperability of these technologies, ensuring that even smaller mining operations can reap the benefits of intelligent maintenance practices.

## 5. Discussion

The main aim of this paper is to conduct a comprehensive review of the existing literature to provide a substantive analysis of the key areas of pump maintenance in relation to the mining industry. A total of 88 articles meeting the established selection criteria were reviewed, allowing for an in-depth examination of the analyzed issue. Such deep analysis makes it possible to answer the stated research questions:

RQ1 intended to discover the state of the literature between 2005 and 2024 on mining equipment maintenance, with a particular focus on pump systems. The main research outputs here are discussed broadly in Section 4.1 and Section 4.2.

The mining equipment maintenance literature, specifically on pump systems, has evolved significantly between 2005 and 2024. Research has primarily concentrated on improving efficiency, reducing downtime, and enhancing predictive maintenance strategies through advanced sensor-based monitoring, AI-driven analytics, and automation technologies.

The reviewed literature has been categorized into six major research areas, each addressing different aspects of pump reliability, performance optimization, and failure prevention.

**Dewatering systems** are essential in both open-pit and underground mining to control water levels, prevent flooding, and ensure smooth operations. The literature extensively discusses the following:

- Optimization of pumping systems—research explores the selection of pump configurations, balancing flow rates, and designing multi-stage pumping stations to enhance water removal efficiency.
- Impact of harsh mining environments—studies highlight the abrasive nature of mine water, which accelerates wear in pump components such as impellers and seals.
- Remote monitoring of dewatering pumps—the use of IoT-enabled sensors and automated diagnostics has been explored to improve operational oversight and reduce manual inspections.

In this area, across multiple studies, the challenge of pump reliability in extreme environments (e.g., high-pressure conditions, variable water loads) emerges as a critical issue. Solutions often involve integrating real-time monitoring with predictive maintenance techniques.

In addition, mining operations depend heavily on the continuous and efficient operation of pumps, making performance optimization a key research area. Indeed, the key contributions in the area of **operational efficiency and reliability optimization** mostly include the following:

- Energy consumption analysis—studies have quantified the energy footprint of pumps in mining operations, emphasizing the potential for efficiency improvements through variable-speed drives (VSDs) and frequency conversion technologies.
- Computational fluid dynamics (CFD) in pump design—CFD has been widely applied to simulate fluid flow, identify turbulence effects, and optimize pump impeller designs.
- Impact of wear on pump performance—research frequently examines degradation mechanisms (e.g., cavitation, erosion, and corrosion), providing insights into material selection and protective coatings.

**Condition/health status monitoring** was the third research area under investigation. The shift towards real-time condition monitoring and predictive maintenance is one of the most notable trends in pump maintenance research. The key insights from the articles in this research area are as follows:

- Early detection of faults: Several studies emphasize the importance of monitoring pump performance to identify potential issues before they result in significant breakdowns or inefficiencies. Predictive techniques help optimize maintenance schedules and minimize unplanned downtime.
- Wear and tear monitoring: Many studies focus on slurry pump wear, particularly the wear of impellers. Wear patterns are influenced by factors like flow rate, particle concentration, and particle size, which help inform better pump design, maintenance, and operational strategies.
- Advanced monitoring methods: The use of diverse methodologies, including unsupervised clustering, multiphase flow models, nodal analysis, and deep learning-based fault detection, is central to these studies. These methods allow for the prediction of pump health and early intervention to prevent failures.
- Real-time and preventive maintenance: t = Technologies such as intelligent electronic devices (IEDs), simulation-based monitoring, and real-time data analysis (e.g., power consumption and pressure measurements) are highlighted as essential for ensuring optimal performance and preventing breakdowns.
- Multisensor and intelligent systems: The integration of multisensor systems in monitoring and the use of intelligent systems, such as IEDs and simulation models, play a crucial role in enhancing preventive maintenance capabilities and ensuring the safety of mining operations.
- Focus on safety: Real-time monitoring of various equipment, including pumps, is critical to ensuring safety in mining operations, particularly in relation to issues like gas leaks, temperature, and pressure monitoring.

In summary, the research underscores the significance of advanced monitoring techniques and real-time data analysis in improving pump reliability, extending lifespan, and ensuring the safety of mining operations.

The fourth research area is connected with **health diagnosis and prognosis**. Health diagnosis involves detecting faults and wear in pumps through real-time monitoring using sensors and diagnostic algorithms. Prognosis, on the other hand, predicts the future health of the pump, estimating its remaining useful life (RUL) and potential failures using data from diagnostic tools and environmental conditions.

The research highlights the growing importance of data-driven approaches such as machine learning, predictive analytics, and condition-based monitoring. These tools enhance diagnostic accuracy and predictive maintenance, helping shift from reactive to proactive maintenance strategies in mining operations. This transition is vital for improving the sustainability and efficiency of the mining industry. Key research areas include the following:

- Prediction of remaining useful life: Advanced models like hidden semi-Markov models, relevance vector machines, and support vector machines have been used to predict the RUL of hydraulic pumps, slurry pump impellers, and other components. These models improve the accuracy of predictions, reducing unplanned breakdowns and optimizing maintenance schedules.
- Condition monitoring and diagnostics: Research has focused on real-time monitoring of pump health, with applications like vibration-based monitoring, thermal imaging, and automated diagnostics using PLC control. These systems aim to identify faults early, preventing failures and extending the operational life of pumps.
- Technological integration and innovation: The incorporation of advanced technologies, including AR, dynamic modeling, and machine learning, has been explored to enhance diagnostic capabilities and predictive maintenance. These innovations allow for more efficient maintenance strategies, particularly in remote and extreme environments like oil rigs and mining operations.
- Health monitoring of specific pump types: Studies have also focused on the health monitoring of specific pump types, such as hydraulic plunger pumps and piston diaphragm pumps, exploring failure mechanisms and proposing solutions to improve reliability and reduce operational costs.

In summary, the research underscores the importance of advanced simulation, predictive analytics, and data-driven maintenance in improving the performance and reliability of pumps in challenging industrial environments. These methods prevent costly downtimes and contribute to cost-effective and sustainable mining operations.

A well-structured maintenance strategy is crucial for cost reduction and equipment longevity. Thus, **maintenance management** in the mining industry, particularly for pump systems, is a critical practice aimed at ensuring the reliable, efficient, and cost-effective operation of pumps used in water management, slurry transport, and hydraulic systems. It involves the integration of preventive, predictive, and condition-based maintenance strategies, which minimize unplanned failures, optimize energy consumption, and extend equipment lifespan.

Research in this area identifies six key studies that explore various aspects of maintenance management for mining pumps, with a focus on automation, data-driven decision-making, and advanced maintenance strategies. These articles collectively demonstrate that effective maintenance management for mining pumps requires a combination of automation, data-driven decision-making, and lifecycle optimization of assets. These studies provide valuable insights into improving maintenance strategies to enhance pump performance, reduce operational risks, and lower costs. Looking ahead, advancements in AI, IoT, and sustainability-focused technologies will further refine maintenance strategies, driving more efficient, cost-effective, and environmentally friendly mining operations.

The last research area refers to **intelligent mining** recognized as the integration of advanced digital technologies to optimize and automate mining operations, enhancing efficiency, safety, and sustainability. This field combines big data analytics, artificial intelligence (AI), the Internet of Things (IoT), cloud computing, automation, robotics, and digital twin technology to enable real-time monitoring, predictive maintenance, and autonomous decision-making in mining operations.

A central aspect of intelligent mining is data-driven decision-making, where IoT sensors and AI algorithms continuously collect and analyze operational data. This approach allows for predictive maintenance strategies that minimize downtime and extend equipment lifespan. Automation plays a crucial role by deploying autonomous vehicles, drilling systems, and robotic machinery, reducing human intervention, particularly in hazardous environments. Additionally, remote monitoring and control enable centralized operation centers to optimize mining processes in real-time.

The transition from reactive maintenance to predictive and proactive maintenance strategies is a defining feature of intelligent mining. By anticipating and preventing issues before they occur, mining companies can achieve more resilient and cost-effective operations. The integration of digital ecosystems enables different mining components—such as pumps, conveyors, drilling equipment, and ventilation systems—to communicate and adapt dynamically, improving overall mine productivity.

In the context of pump maintenance, intelligent mining transforms traditional approaches by introducing real-time condition monitoring, AI-based fault detection, and automated control systems. IoT-enabled sensors track pump performance, while AI-driven models predict potential failures before they occur, reducing unplanned downtime. Digital twin technology further enhances maintenance strategies by simulating pump behavior under various conditions, enabling optimized operational planning. These advancements ensure higher reliability, reduced maintenance costs, and improved operational efficiency.

The research identified four key papers in intelligent mining, specifically focusing on pump maintenance. These papers cover various aspects of intelligent pump system management in the mining industry, highlighting the use of technologies such as big data analysis, automation, adaptive control, and digital ecosystems. All the studies aim to improve the reliability and efficiency of pump systems through intelligent monitoring, fault diagnosis, and operational optimization.

Ultimately, intelligent mining reshapes pump maintenance by combining automation, predictive analytics, and real-time data processing. The ability to anticipate failures before they occur, automate routine operations, and interconnect mining infrastructure leads to a more resilient, cost-effective, and sustainable industry.

The conducted systematic analysis of the selected literature makes it possible to answer the second research question. RQ2 intended to define the main research and knowledge gaps in pump systems maintenance, especially in the mining industry sector. Several key gaps can be highlighted based on the literature reviewed in the context of pump maintenance (presented in Section 4).

The first gap is the aspect of integration of advanced predictive models. While recent advancements in data-driven models, artificial intelligence (AI), and the Internet of Things (IoT) for predictive maintenance have made significant progress, there is still a gap in integrating more advanced predictive models applied in the mining industry. Current approaches often focus on monitoring basic pump parameters and do not consider the full range of influencing factors, such as environmental variables (e.g., temperature, humidity) or detailed data from other systems within the mine. There is a need for the development of more advanced AI algorithms that take into account a broader operational context and can predict failures more precisely. This would help minimize downtime and repair costs by identifying potential issues before they arise and improving the accuracy of maintenance strategies.

Next, comprehensive automation solutions development. Automation is a key element in improving the efficiency of pump systems in mining; however, there are still gaps in fully automating pump operations in the most challenging, often remote or hazardous locations within mines. While some technologies enable remote control, many systems still require human intervention in critical stages. There is a need for further development in autonomous systems that can manage the entire lifecycle of pump operations, including automated fault detection, performance optimization, and self-adjustment based on varying environmental conditions. Expanding automation capabilities would help reduce the dependence on human labor in high-risk areas and improve overall operational efficiency.

Data-driven maintenance strategies for complex systems are another area of research interest worth further development. While condition-based and predictive maintenance strategies have been widely applied to pumps, many mining operations still lack comprehensive systems for data-driven maintenance across complex systems. Pump systems in mining are often interconnected with other critical systems, such as conveyors, hydraulic equipment, and ventilation systems, and maintaining them effectively requires considering the interactions and dependencies between these systems. Future research should focus on developing integrated data ecosystems that allow for real-time monitoring, fault diagnosis, and predictive maintenance of pumps in conjunction with other equipment. This would lead to a more holistic and efficient approach to maintenance, improving overall mine productivity and reducing unplanned downtime.

According to recent developments, one should also focus on the problem of digital ecosystems scalability. The concept of digital ecosystems, where networks of sensors, pumps, and control systems interact dynamically, is still in its infancy in mining operations. While localized pump systems can be monitored and controlled remotely, scaling these systems to operate across multiple mine sites remains a challenge. More research is needed into scalable digital ecosystem architectures that enable seamless communication and integration between individual systems at a global level. This would allow for centralized control, predictive maintenance, and real-time decision-making across a mining company’s entire infrastructure, improving coordination and operational efficiency across various sites.

Additionally, one of the research gaps is environmental and external influences on pump performance investigation. Environmental factors such as temperature, humidity, and even the chemical composition of the fluid being pumped can have significant impacts on pump performance and lifespan. However, many current maintenance strategies do not fully account for these variables. Future research could explore how to integrate environmental data into predictive maintenance models, taking into consideration how these factors affect pump performance. This would lead to more tailored and effective maintenance strategies that extend the operational lifespan of pumps and reduce failures caused by overlooked external influences.

The last research gap that could be identified in the investigated research area is the focus on human factors and user-centered maintenance. Despite the rapid advancement of automation, AI-driven diagnostics, and predictive maintenance strategies, human factors remain a crucial yet often underexplored element in the maintenance of pump systems [188,189]. While modern technologies enable real-time monitoring and data-driven decision-making, human operators continue to play a key role in interpreting diagnostics, executing maintenance actions, and responding to unforeseen system behaviors. Integrating proactive maintenance approaches introduces several challenges related to human factors, which must be addressed to ensure their effective implementation [190,191]. The problem is worth investigating, as we can see from the mining accident causality, where human error is one of the most significant causes (see, e.g., [7,192]). In addition, a comprehensive analysis of human errors in pump maintenance is given in [193].

One of the key challenges is the growing skills gap among maintenance personnel. The increasing complexity of predictive maintenance tools, such as IoT-enabled monitoring systems and AI-based diagnostics, requires specialized knowledge that is not always covered in traditional training programs. Without adequate preparation, maintenance staff may struggle to interpret diagnostic outputs correctly, leading to inefficiencies in maintenance execution and potential equipment failures. Additionally, adopting advanced maintenance technologies often encounters resistance from employees who are unfamiliar with digital tools or uncertain about their reliability. A lack of trust in automated decision-making systems can hinder their widespread use, emphasizing the need for strategies that enhance confidence in AI-assisted diagnostics.

Another critical issue is the cognitive load imposed on operators who must process vast amounts of diagnostic data from multiple sources. Poorly designed human-machine interfaces (HMI) can lead to information overload, increasing the likelihood of errors and reducing the effectiveness of predictive maintenance strategies. Ensuring that diagnostic tools present data clearly and intuitively is essential for enabling quick and informed decision-making. Furthermore, while AI-driven systems can generate predictive insights, human expertise remains indispensable in validating recommendations and making final maintenance decisions. The absence of well-defined interaction models between human operators and automated maintenance systems can limit the effectiveness of proactive maintenance approaches.

The concept of Maintenance 5.0 introduces a more human-centric approach in which automation and AI do not replace human expertise but rather enhance it. However, research on how to effectively integrate human decision-making with automated diagnostic tools in the context of mining pump maintenance remains limited. Addressing this gap requires a stronger focus on workforce training, user-friendly interface design, and hybrid decision-making frameworks that combine human intuition with AI-driven analytics.

To fully realize the potential of proactive maintenance, future research should explore strategies for improving the skills of maintenance personnel, developing intuitive interfaces that minimize cognitive overload, and fostering trust in AI-assisted maintenance tools. Implementing Maintenance 5.0 principles in pump maintenance should prioritize human-system collaboration, ensuring that operators remain central to the decision-making process while benefiting from the efficiency and precision offered by automation. By incorporating human factors into predictive maintenance strategies, organizations can enhance pump systems’ reliability and operational efficiency, ultimately contributing to safer and more resilient mining operations.

In summary, these knowledge gaps represent opportunities for innovation in the field of pump system maintenance in the mining industry. Advancing predictive maintenance models, automating more complex systems, integrating environmental factors, and expanding digital ecosystem scalability will drive the next generation of maintenance strategies, leading to more efficient, cost-effective, and sustainable mining operations.

RQ3 is intended to discover the main trends that can be identified in proactive maintenance approaches and how they have evolved over recent years in the mining sector. According to the conducted literature review, the main trends in proactive maintenance approaches in the mining sector (pump maintenance) and their evolution in recent years reflect the growing integration of advanced technologies, data-driven decision-making, and automation. Table 4 provides a structured comparison of key research areas’ current trends and challenges, offering a clear roadmap for future research in mining pump maintenance.

Over the past few years, proactive maintenance approaches in the mining sector have undergone significant transformation, driven by advancements in technology, data-driven decision-making, and automation. Traditionally, mining maintenance was largely reactive, relying on repairs and interventions after equipment failures. However, there has been a marked shift towards predictive and proactive maintenance strategies, which focus on anticipating problems before they occur, reducing downtime, and extending equipment lifespan.

Following Table 2, one of the key trends is the movement from reactive maintenance to predictive and proactive approaches. In the past, maintenance was often based on scheduled inspections or took place after failures occurred, leading to inefficiencies. With the rise of predictive maintenance powered by data analytics and machine learning, maintenance is now driven by data collected from equipment sensors and historical performance. This allows operators to predict when maintenance is needed, enabling them to act before problems arise. Using AI and machine learning models enhances the accuracy of these predictions, providing more reliable and effective maintenance strategies.

Another major trend is the increasing use of the Internet of Things (IoT) and real-time monitoring systems. IoT-enabled sensors are now widely used to track critical parameters such as real-time temperature, pressure, and vibration of mining equipment. These data are continuously analyzed, allowing operators to identify potential failures early, optimize maintenance schedules, and improve operational efficiency. Real-time monitoring has become essential for maintaining the health of mining equipment, reducing unplanned downtime, and ensuring a more resilient operation.

Integrating AI and machine learning in maintenance decision-making is also a key development. Traditional maintenance approaches were often based on simple inspections or time-based schedules, which sometimes missed early signs of potential failures. With AI, mining companies can now rely on data-driven models to anticipate equipment failures, optimize schedules, and ensure that maintenance is performed only when needed rather than following a fixed schedule. This shift significantly reduces unnecessary downtime and maintenance costs.

Automation and autonomous systems have also played a crucial role in evolving maintenance strategies. In the past, maintenance tasks were largely manual, requiring human intervention for inspections and repairs. Today, autonomous systems, such as robotic maintenance platforms and drones, can conduct inspections, perform routine maintenance tasks, and even diagnose faults in hazardous environments. This improves operational efficiency and enhances safety by reducing the need for human workers in dangerous areas.

Digital twin technology is another important trend that has emerged in proactive maintenance. A digital twin is a virtual representation of physical assets, such as pumps or machinery, enabling real-time monitoring and simulation of equipment performance. By creating a digital replica of the equipment, mining companies can simulate various operating conditions and predict potential failures before they occur. This allows for better planning, optimized maintenance schedules, and improved equipment performance.

Big data analytics and data-driven decision-making have become integral to proactive maintenance approaches. In the past, maintenance decisions were often based on limited data or subjective assessments. Mining companies can now make more informed decisions by integrating sensor data, historical performance records, and environmental conditions. Big data analytics help identify subtle patterns that may indicate potential issues, allowing for timely interventions that prevent breakdowns and optimize equipment performance.

Sustainability and energy efficiency are also increasingly influencing maintenance strategies. As environmental concerns grow, mining companies focus on reducing energy consumption and extending the lifespan of equipment. Proactive maintenance now includes monitoring energy usage, identifying inefficiencies, and taking steps to reduce energy waste. This aligns with broader sustainability goals and helps minimize mining operations’ environmental impact.

In summary, proactive maintenance strategies in the mining sector have evolved significantly, shifting from reactive approaches to more predictive and data-driven models. Integrating advanced technologies such as IoT, AI, machine learning, automation, and digital twins has improved maintenance efficiency, reliability, and cost-effectiveness. These trends enhance the overall performance of mining operations and contribute to more sustainable and energy-efficient practices.

The conducted systematic analysis of the selected literature makes it possible to answer the last research question. RQ4 is intended to define the framework’s scope for proactive maintenance of pump systems in the mining industry.

The proactive maintenance framework for pump systems in the mining industry aims to enhance mining operations’ reliability, efficiency, and sustainability by utilizing advanced digital technologies for maintenance management. The framework’s scope should be built on integrating real-time data monitoring, predictive analytics, and performance optimization strategies to ensure continuous, efficient, and cost-effective operations.

Key elements that should be included in the framework are as follows:

1. Real-Time Data Acquisition and Integration:
  - Teal-time data collection through IoT sensors embedded in the pump systems is a critical component. Data from these sensors will provide a continuous flow of information on pump health, operational performance, and environmental factors.
  - Data must be integrated from various sources, including maintenance history, sensor data, operational logs, and environmental conditions, to comprehensively view the pump system’s performance.
2. Predictive Maintenance Algorithms:
  - The core of the framework is the implementation of predictive maintenance strategies. Using machine learning models and statistical analysis, these algorithms will predict potential failures before they occur, allowing the operators to schedule maintenance tasks proactively.
  - Predictive maintenance will reduce unplanned downtimes, enhance the lifespan of pumps, and optimize resource allocation.
3. Data Analytics and Visualization:
  - The framework should include advanced data analytics tools to identify patterns and anomalies in the operational data, providing actionable insights.
  - Visualization dashboards will allow operators and maintenance personnel to easily interpret data, spot emerging issues, and make informed maintenance and operational adjustments decisions.
4. Simulation and Scenario Testing:
  - Simulation models of the pump systems will enable the testing of various maintenance strategies and failure scenarios. This allows the mining operators to evaluate the effectiveness of different approaches without disrupting actual operations.
  - This component also aids in optimizing maintenance schedules and identifying the best timing for interventions to minimize the impact on overall productivity.
5. Condition Monitoring and Performance Tracking:
  - Continuous monitoring of the pump systems, including key parameters such as pressure, temperature, flow rate, and vibration levels, will provide real-time insight into their operational health.
  - Automated condition monitoring tools will send alerts for any deviations from normal operating conditions, allowing quick actions to be taken before a potential failure occurs.
6. Feedback Mechanisms for Continuous Improvement:
  - The framework should incorporate feedback loops that capture the outcomes of maintenance activities and compare them with the predictions made by the system. By continually analyzing maintenance results, organizations can refine their predictive models and improve maintenance strategies.
  - A continuous improvement process helps the organization enhance the accuracy and effectiveness of its predictive maintenance capabilities.
7. Collaboration and Communication Tools:
  - Efficient communication tools are essential for coordinating maintenance activities, especially in complex mining operations. The framework should include collaboration platforms that allow seamless information sharing between maintenance teams, management, and operators to ensure that all stakeholders are informed about the pump systems’ status and any required actions.
8. Compliance and Reporting:
  - The framework must ensure compliance with industry standards and regulatory requirements for maintenance practices. It should include reporting functionalities to generate maintenance logs, compliance reports, and performance reviews.
  - The data-driven approach will help demonstrate environmental, safety, and operational standards adherence.
9. Scalability and Adaptability:
  - The framework should be designed to be scalable, supporting various pump systems used in mining operations of different sizes and complexities.
  - It should also be adaptable to future technological advancements, ensuring that the system can evolve with emerging technologies such as 5G communications, AI, and enhanced data analytics tools.

As the analysis outlines, the proactive maintenance framework for pump systems in the mining industry should focus on integrating advanced digital technologies, such as IoT, data analytics, predictive maintenance, and real-time monitoring. Additionally, this framework should minimize downtime, reduce maintenance costs, and enhance operational efficiency, ultimately improving asset longevity and sustainability in mining operations.

By incorporating these core elements, the framework will maintain pump systems proactively, mitigating risks and avoiding unplanned failures. It also aligns with the broader industry trend of moving from reactive maintenance strategies to more sophisticated, data-driven, and predictive approaches.

## 6. Framework for Proactive Maintenance in Pump Systems from the Mining Industry

After ventilation systems, pumping systems are the most important elements of mine operation. They ensure the safety of workers and allow for the proper conduct of work in the mine. For example, pumping stations for the main drainage system must allow water to be continuously removed from mine workings regardless of emergency conditions or the suspension of mining operations. Two independent sources of electricity must power systems of this type. The control of such pumps should allow switching between pumps automatically or manually. Depending on the requirements and design of these systems, various measurements of operating parameters are used, i.e., capacity, water temperature, bearing temperature, supply voltage, motor supply current, head, and operating pressure. Depending on these indications, pumps are adjusted, or diagnostic and maintenance activities are undertaken.

Determination of the unreliability of pumping systems largely depends on the technical evaluation of electrical, mechanical, or electromechanical parameters. At the same time, one of the key problems in the operation of mining pumps is correctly identifying the first symptoms of impending failure and making economically rational operating decisions. Due to the issue’s complexity and the difficult operating conditions of the pumps, proper management of mining pump maintenance should be based on a proactive approach using a predictive strategy.

**Figure 24:**
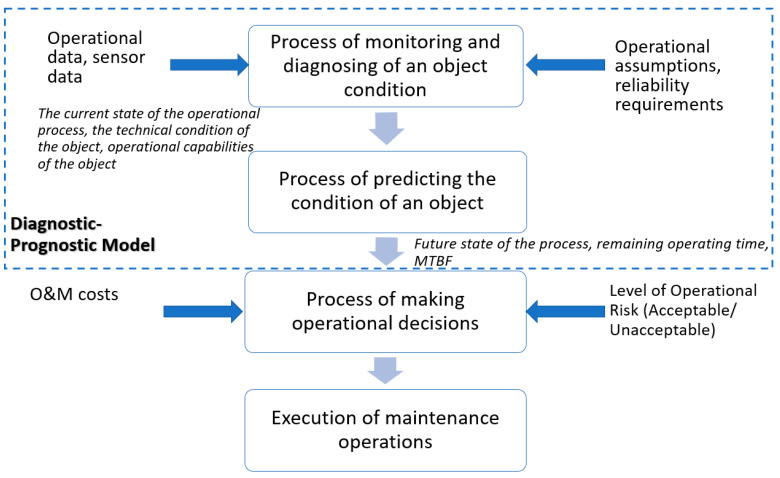
Proposed proactive approach in mining pump maintenance.

The general concept of the proactive approach in mining machinery maintenance, proposed by the authors, is shown in Figure 24. It includes two basic elements—the development of a diagnostic-prognostic model and the definition of stages in making operational decisions based on the determined level of operational risk and the incurred operational costs. It was developed for the pumps performing in the selected Polish mine. Preliminary studies also made it possible to determine the conditions of applicability of the various detection methods and refine the plan for in-service testing to ensure the efficiency of the diagnostic work carried out (see [129]). In practice, this means that mining operators in the selected mine now use a diagnostic model developed through the insights from the literature review to assess pump conditions. The implementation of predictive maintenance strategies allows for better resource allocation, timely detection of anomalies, and more informed decision-making regarding equipment repairs and replacements.

The first step in the implementation of the proactive maintenance framework should focus on condition forecasting and rational decision-making processes for operations. This entails refining assumptions and requirements based on detailed diagnostic testing, such as vibroacoustic diagnostics, acoustic tests, and thermal measurements. The designed solution assumes the following operational conditions:

Cascade shaft drainage system:

- Pumps operating in cascade should be responsible for transporting water from different depth levels to the surface.
- The system should allow automatic start-up of subsequent pumps in the event of failure of one of the lower level pumps to avoid interruption of dewatering.
- Specific Recommendation: Implement an IoT-based monitoring system to continuously assess pump health (vibration, temperature, etc.) and trigger automatic start-up of backup pumps when necessary.

Surface pumps:

- Pumps on the surface responsible for removing process water to the main pipeline must work reliably, as their downtime could lead to flooding of the mine.
- In case of pump overload, the system should automatically switch to another pump to ensure continuous operation.
- Specific Recommendation: Use vibration and thermal sensors to monitor real-time operational conditions and integrate these with an automated switching mechanism that activates alternative pumps when needed.

Proactive diagnostics:

- The maintenance system should use various diagnostic methods to monitor the condition of pumps, including vibroacoustic diagnostics, acoustic tests, and thermal measurements that have been pre-tested.
- Monitoring of the condition of rolling elements should be regular and allow detection of anomalies at an early stage.
- Specific Recommendation: Develop a predictive maintenance dashboard that integrates all sensor data to provide early warnings for potential failures, allowing for a data-driven, proactive response.

Requirements for the proactive maintenance method are presented in Table 5.

Additionally, it is necessary to define the predictive analysis assumptions to accurately predict future failures, enabling maintenance planning in a way that minimizes production disruption and extends equipment life.

At the same time, correct inference requires summarizing the process of evaluating the technical condition of pumps and their components based on accumulated diagnostic test results. This includes analysis of historical failures and diagnostic results, such as vibroacoustic or thermal measurements. To this end, a set of diagnostic indicators (e.g., RMS vibration level and bearing temperature) was proposed to assess the technical condition of pumps. In the next step, permissible values and alarm thresholds (for normal, warning, and critical conditions) should be proposed as a reference for evaluating the measurement results. The cooperating entity is currently verifying the alarm thresholds.

A classification of damage types based on diagnostic tests must be developed to prepare a proactive maintenance method.

All these aspects should be prepared according to the operational and technical conditions of the given mining company.

The final component of the method is the operational decision-making process. In the developed decision-making process based on the diagnostic-predictive model, recommendations include different types of recommendations adapted to the characteristics of detected anomalies, failure predictions, and the overall condition of the machine. The recommendations are initially divided into eight categories: recommendations for preventive services, recommendations for corrective operational measures, recommendations for emergency repairs, recommendations for automatic actions, recommendations for resources and materials, recommendations for optimizing the operation process, long-term recommendations, and recommendations for security. These recommendations should be tailored to the specific operation of the machine or equipment in question, considering current operating conditions and the long-term goals of optimizing the operation process.

In addition, based on the findings of the literature review and referring to the defined framework, mining companies can implement the following specific steps to enhance pump maintenance practices:

- Use of vibroacoustic diagnostics, acoustic tests, and thermal measurements to continuously monitor the condition of pumps;
- Development of a predictive analytics model that considers pump characteristics and operational loads to forecast remaining operating time before failure;
- Implementation of an automatic response system that triggers alarms and initiates corrective actions (e.g., switching pumps or shutting them down remotely in case of critical anomalies);
- Establishment of a service scheduling system that adapts to the real-time condition of the pumps (i.e., “maintenance on condition”);
- Integration of these diagnostic and predictive techniques into the mine’s central management system, allowing operators to make real-time decisions regarding maintenance and operational strategies.

## 7. Conclusions

This article presents a systematic literature review focused on the proactive maintenance of pump systems in the mining industry. By analyzing key publications and case studies from 2005 to 2024, the review provides a comprehensive overview of the evolving approaches and best practices for maintaining pump systems in the mining sector. This study aimed to identify the main trends, research gaps, and emerging methodologies in proactively maintaining these critical assets.

The findings highlight the increasing importance of predictive maintenance strategies, the integration of digital technologies such as IoT and data analytics, and the role of real-time monitoring in improving operational efficiency and reducing downtime. Additionally, the research identifies the growing trend of leveraging simulation models and AI-driven algorithms for forecasting equipment failures and optimizing maintenance schedules.

However, the study has several limitations. The scope of the review is primarily focused on pump systems within the mining sector, which may exclude valuable insights from other industries or related sectors that employ similar maintenance strategies. The literature search and analysis were mostly restricted to peer-reviewed articles and conference papers, which might have overlooked significant contributions from industry reports, technical papers, or other non-academic sources. Furthermore, the research methodology did not include a quality assessment of the reviewed publications based on citation counts or relevance, which could affect the generalizability of the findings.

The review also highlights that although significant progress has been made in applying proactive maintenance strategies in mining, there are still gaps in understanding the full potential of emerging technologies, such as digital twins, in this specific context. The integration of advanced predictive maintenance systems, the development of standardized frameworks, and the establishment of industry-wide best practices are key areas requiring further research. Additionally, while this study provides a technical perspective on proactive maintenance, it does not fully address the human factors involved in its implementation, such as staff training, the adoption of new technologies, and organizational readiness. These factors play a crucial role in the success of proactive maintenance programs, and future research should consider these aspects to provide a more comprehensive approach to maintenance system implementation.

In conclusion, the study emphasizes the need for continued advancements in maintenance management practices, particularly in terms of embracing digital transformation and incorporating innovative technologies to improve asset reliability and performance. Future research should focus on exploring the challenges and opportunities of implementing these frameworks in real-world mining operations, addressing issues such as system integration, data security, and scalability. Moreover, further studies are needed to investigate the environmental and economic impacts of adopting proactive maintenance strategies and the potential for new business models that leverage emerging technologies in the mining sector.

## Figures and Tables

**Table 1:** A summary of recent papers focusing on providing a literature overview in the area of pump maintenance.

Ref.	Publication Year	Research Objectives	Methodology Used	Databases Analyzed	Papers Analyzed	Analyzed Sector or Type of Pumps
[73]	2011	Review on major fault modes in centrifugal pumps, especially in the context of the water and sewage industry applications.	n/a	n/a	n/a	Water and sewage industry/Centrifugal pumps
[74]	2014	Review on diagnostics and prognostics of centrifugal pumps, focusing on the application area, detection of fault modes, and test results.	n/a	n/a	n/a	Centrifugal pumps
[33]	2014	A comprehensive review of the development, applications, and performance characteristics of pumps operating in turbine mode (PAT) for small-scale hydroelectric power systems, with a focus on their feasibility in remote communities lacking grid connections.	n/a	n/a	n/a	Pump as turbine (PAT)
[34]	2020	Review on prognostics and health management for pump systems operating in thermal power plants.	Structured overview	n/a	n/a	Energy sector
[35]	2021	Analysis and characteristics of faults in large-scale heat pumps, with particular attention to recurrent faults in critical components, to assess existing fault detection and diagnosis methods, identify their limitations, and propose future research directions.	n/a	n/a	n/a	Large-scale heat pumps
[75]	2022	Critical analysis of machine learning’s most current advances in the field of artificial intelligence-based system health management.	Systematic literature review	Web of Science, Science Direct, Scopus	88	Oil and gas sector
[61]	2022	Review various types of faults and their identification possibilities by various traditional and ML-based techniques.	n/a	n/a	n/a	Centrifugal pumps
[76]	2022	Investigation of the potentials and challenges of integrating emerging technologies (digital twin, building information modeling, machine learning) in the PdM of pumps.	Systematic literature review	Web of Science, Scopus	118	Civil infrastructure applications
[77]	2022	Review on condition monitoring techniques for pumps with a particular focus on the sensing technologies.	n/a	n/a	n/a	Oil and gas sector
[78]	2022	Review on machine learning-based fault diagnosis of centrifugal pumps.	n/a	n/a	n/a	Centrifugal pumps
[79]	2022	Review on recent developments in hydraulic pumps’ fault diagnosis and health management.	Systematic literature review	n/a	n/a	Hydraulic pumps
[80]	2022	Review on health monitoring methods suitable for airframe fuel pumps under flight conditions.	n/a	n/a	n/a	Aviation sector
[81]	2023	Review on the artificial intelligence (AI)-based models for PdM of water injection pumps.	Systematic literature review	Scopus, IEEE Xplore, ScienceDirect, and ACM Digital Library	16	Oil and gas sector
[82]	2024	General overview of control systems used to operate pump units.	n/a	n/a	n/a	Offshore industry
[83]	2024	Review of the centrifugal pump cavitation monitoring methods (flow-head method, high-speed photography, pressure pulsation method, acoustic emission method, vibration method).	n/a	n/a	n/a	Centrifugal pumps

n/a—not available.

**Table 2:** Comparison of approaches in pump health diagnosis and prognosis.

Approach/Method	Ref.	Technology/Model	Advantages	Disadvantages	Contradictions/Inconsistencies in Findings
Support Vector Machines (SVMs)	[52]	SVM for RUL assessment	Effective with sensor data analysis, good for damage identification	Requires large amounts of data and well-tuned parameters	Does not perform well with low-frequency measurements
Segmental Hidden Semi-Markov Models (HSMMs)	[160]	Hidden semi-Markov model for RUL prediction	Improved accuracy in RUL prediction, better state detection in pumps	High computational requirements, challenging implementation in large systems	Effective on field data, but not always reliable in unstable operational conditions
Relevance Vector Machines (RVMs)	[161]	RVM for RUL prediction	More accurate than traditional methods, lower data requirements	Needs precise feature selection, sensitive to data errors	Results can vary depending on operational conditions
ARIMA	[163]	ARIMA model for RUL prediction	Simple to apply, effective for time series data	Low effectiveness with irregular data, parameter tuning required	Inconsistent results across different pump systems
Vibration-based Monitoring	[164]	Vibration monitoring for failure detection	Early damage detection, cost-effective	May not detect all types of failure, needs specialized sensors	Results vary depending on background vibration in different environments
Augmented Reality (AR) for Diagnostics	[168]	AR technology for diagnostics	Enables real-time diagnosis, supports technicians	High implementation cost, requires advanced infrastructure	Sometimes insufficient data for full analysis in field conditions

**Table 3:** Comparison of technological approaches for intelligent pump maintenance in mining operations.

Aspect	Approach 1 (Big Data and Machine Learning)	Approach 2 (IoT and 5G for Remote Monitoring)	Approach 3 (Automation and Digital Twin)	Approach 4 (PLC and Automation)
Technologies	Big data, machine learning, fault prediction	IoT, 5G, remote monitoring, automation	IoT, digital twin, operational condition analysis	PLC, automation, real-time monitoring
Advantages	High accuracy in fault detection, autonomous diagnostics	Remote control, real-time monitoring, low operational costs	Optimized planning, predictive failure analysis	Reduces manual labor, easy to integrate with existing systems
Disadvantages	High data quality requirements, integration challenges	High initial cost, integration issues with legacy systems	High technology requirements, incompatibility with older equipment	Limited scalability, dependency on PLC infrastructure
Applications	Fault diagnosis, predictive maintenance using data models	Real-time monitoring, fault detection and control	Simulation and optimization of pump operation	Automation in water intake systems, pump rotation control
Infrastructure Compatibility	Challenging integration with legacy systems	Difficult adaptation for older systems	High technological demands, not always compatible with older setups	Easy integration with existing PLC systems
Scalability	Limited by data availability, dependent on large data sets	Suitable for remote operations, limited local control	High infrastructure demands, scalability challenges	Scalability depends on PLC size and budget
General Comparison	Effective in predictive fault diagnosis and autonomous decision-making	Focuses on remote monitoring and real-time system adjustments via IoT and 5G	Emphasizes automation and optimization using simulations (digital twins) for maintenance and control	Focuses on basic automation using PLC systems and sensors for real-time fault detection and control
Example References	[185]	[186]	[183,184]	[66]

**Table 4:** A summary of the main trends and challenges identified in mining pump maintenance (2005–2024).

Research Area	Main Trends	Challenges
Dewatering System Operation and Maintenance	- Increasing use of automated water management - Integration of IoT-enabled sensors for real-time monitoring - AI-driven predictive dewatering models	- Abrasive and corrosive conditions reducing pump lifespan - Need for real-time system adjustments under changing mine conditions - High cost of automated dewatering solutions
Operational Efficiency and Reliability Optimization	- Adoption of variable-speed drives (VSDs) to optimize energy use - AI-based pump performance optimization - Material science advancements to reduce wear	- High energy costs of pumping operations - Need for customized CFD models for different pump types - Complex relationship between fluid dynamics and wear mechanisms
Condition/Health Status Monitoring	- Widespread use of sensor-based monitoring - Integration of cloud-based diagnostics - AI-driven failure prediction models improving accuracy	- Managing large-scale sensor data efficiently - False positives in anomaly detection - Standardization issues in monitoring technologies
Health Diagnosis and Prognosis	- Use of relevance vector machines (RVMs), hidden semi-Markov models (HSMMs), and support vector machines (SVMs) for RUL estimation - Combining historical failure data with real-time analytics - AI-powered predictive maintenance platforms	- Data quality and availability issues for AI training - Variability in failure patterns under different operating conditions - High computational costs for real-time RUL modeling
Maintenance Management	- Shift from preventive to predictive maintenance - Widespread adoption of computerized maintenance management systems (CMMS) - AI-assisted fault classification for automated decision-making	- Resistance to the adoption of AI-driven maintenance in traditional mining setups - Lack of standard frameworks for maintenance cost analysis - High initial investment costs
Intelligent Mining and Digital Transformation	- Increasing adoption of digital twins for failure prediction - AI-driven predictive analytics becoming standard - Remote and autonomous mining operations reducing human intervention	- Data security concerns with cloud-based diagnostics - Integration challenges with legacy pump monitoring systems - High costs of AI-based automation and digital twin implementation

**Table 5:** Requirements for the proactive maintenance method adopted for the case mining company.

Defined Area	Main Requirements
Monitoring and diagnostics module	The system must be equipped with sensors to measure vibration, noise, and temperature, allowing real-time data collection; these sensors should be installed on critical components (e.g., motor bearings, pump casing) to ensure accurate, real-time condition monitoring. The system must analyze trends in diagnostic data to predict failures, such as from increasing vibration and temperature levels. Actionable Step: Calibrate sensors regularly to ensure data reliability, and integrate them into a centralized system for continuous analysis.
Predictive analytics	The predictive analysis should consider the characteristics of each pump (cascade and surface) and their operating loads, which will allow a more accurate prediction of the operating time remaining before failure. Actionable Step: Develop a machine learning-based predictive model that learns from past pump failures and operational data to more accurately forecast the remaining life of each component.
Automatic response to alarms	The system should generate automatic alarms when the operating parameters of the pumps exceed the established safety thresholds (e.g., temperature of rolling elements or vibration level). In case of critical anomalies, it should be possible to remotely turn the pump off and on or switch it to standby operation. Actionable Step: Implement an automated shutdown mechanism to safely stop pumps if critical thresholds are exceeded and to prevent further damage to the equipment.
Service Scheduling	The system should automatically generate maintenance schedules based on real-time pump data. These schedules should reflect the true condition of the pumps rather than fixed intervals. Service should include regular inspection of rolling elements and lubrication and replacement of components as needed. Actionable Step: Create a dynamic scheduling system that adjusts the maintenance frequency based on wear patterns detected through sensor data (e.g., more frequent inspections for pumps with higher vibration levels).
Reporting and historical analysis	The system should generate detailed reports on pump operation and failures occurring to enable analysis of the causes of failures and optimization of maintenance activities. Reports should also include recommendations for maintenance strategy changes based on historical data analysis. Actionable Step: Use a dashboard for real-time data visualization, allowing engineers to access historical trends and failure analysis quickly, thus enabling more effective decision-making.
Integration with the mine management system	The system should be integrated with the mine’s central management system, allowing real-time monitoring of pumps. Visualizing pump operation and alarms at the management level should be possible so mine operators have full control over the dewatering system. Actionable Step: Set up cloud-based connectivity that allows the central control room to monitor pump health and operation remotely, enabling quick response in case of system failure.

## Data Availability

Not applicable.

## Supplementary Materials

The following supporting information can be downloaded at https://www.mdpi.com/article/10.3390/s25082365/s1: Table S1: The PRISMA Checklist.

## Abbreviations

The following abbreviations are used in this manuscript:

AI	artificial intelligence
AR	augmented reality
ARIMA	auto-regressive integrated moving average
BNN	backpropagation neural network
CBM	condition-based maintenance
CFD	computational fluid dynamics
CM	condition monitoring
CMMS	computerized maintenance management systems
CR	corrective maintenance
DeT	decision tree
DT	digital twin
ESP	electrical submersible pump
FSI	fluid-structure interaction
GAMM	graphical analysis for maintenance management
HSMM	hidden semi-Markov model
HSM	health status monitoring
HVAC	heating, ventilation, air conditioning
IEDs	intelligent electronic devices
IMs	induction motors
IoT	Internet of Things
LiDAR	light detection and ranging
MED	minimum entropy deconvolution
ML	machine learning
MoASoID	method of areas selection of image differences
MTBF	mean time between failures
MTTF	mean time to failure
MTTR	mean time to repair
NN	nearest neighbor
O&M	operation and maintenance
PAT	pump as turbine
Pdis	pump discharge pressure
PdM	predictive maintenance
PIP	pump intake pressure
PM	preventive maintenance
PRISMA	Preferred Reporting Items for Systematic Reviews and Meta-Analyzes
RBFNN	radial basis function neural networks
RVM	relevance vector machine
RUL	residual lifetime
SLR	systematic literature review
SVM	support vector machines
WPD	wavelet packet decomposition
